# iRegulon: From a Gene List to a Gene Regulatory Network Using Large Motif and Track Collections

**DOI:** 10.1371/journal.pcbi.1003731

**Published:** 2014-07-24

**Authors:** Rekin's Janky, Annelien Verfaillie, Hana Imrichová, Bram Van de Sande, Laura Standaert, Valerie Christiaens, Gert Hulselmans, Koen Herten, Marina Naval Sanchez, Delphine Potier, Dmitry Svetlichnyy, Zeynep Kalender Atak, Mark Fiers, Jean-Christophe Marine, Stein Aerts

**Affiliations:** 1 Laboratory of Computational Biology, KU Leuven Center for Human Genetics, Leuven, Belgium; 2 Laboratory for Molecular Cancer Biology, KU Leuven Center for Human Genetics, Leuven, Belgium; 3 VIB Center for the Biology of Disease, Laboratory for Molecular Cancer Biology, Leuven, Belgium; Columbia University, United States of America

## Abstract

Identifying master regulators of biological processes and mapping their downstream gene networks are key challenges in systems biology. We developed a computational method, called iRegulon, to reverse-engineer the transcriptional regulatory network underlying a co-expressed gene set using *cis*-regulatory sequence analysis. iRegulon implements a genome-wide ranking-and-recovery approach to detect enriched transcription factor motifs and their optimal sets of direct targets. We increase the accuracy of network inference by using very large motif collections of up to ten thousand position weight matrices collected from various species, and linking these to candidate human TFs via a *motif2TF* procedure. We validate iRegulon on gene sets derived from ENCODE ChIP-seq data with increasing levels of noise, and we compare iRegulon with existing motif discovery methods. Next, we use iRegulon on more challenging types of gene lists, including microRNA target sets, protein-protein interaction networks, and genetic perturbation data. In particular, we over-activate p53 in breast cancer cells, followed by RNA-seq and ChIP-seq, and could identify an extensive up-regulated network controlled directly by p53. Similarly we map a repressive network with no indication of direct p53 regulation but rather an indirect effect via E2F and NFY. Finally, we generalize our computational framework to include regulatory tracks such as ChIP-seq data and show how motif and track discovery can be combined to map functional regulatory interactions among co-expressed genes. iRegulon is available as a Cytoscape plugin from http://iregulon.aertslab.org.

## Author Summary

Gene regulatory networks control developmental, homeostatic, and disease processes by governing precise levels and spatio-temporal patterns of gene expression. Determining their topology can provide mechanistic insight into these processes. Gene regulatory networks consist of interactions between transcription factors and their direct target genes. Each regulatory interaction represents the binding of the transcription factor to a specific DNA binding site near its target gene. Here we present a computational method, called iRegulon, to identify master regulators and direct target genes in a human gene signature, i.e. a set of co-expressed genes. iRegulon relies on the analysis of the regulatory sequences around each gene in the gene set to detect enriched TF motifs or ChIP-seq peaks, using databases of nearly 10.000 TF motifs and 1000 ChIP-seq data sets or “*tracks*”. Next, it associates enriched motifs and tracks with candidate transcription factors and determines the optimal subset of direct target genes. We validate iRegulon on ENCODE data, and use it in combination with RNA-seq and ChIP-seq data to map a p53 downstream network with new predicted co-factors and targets. iRegulon is available as a Cytoscape plugin, supporting human, mouse, and Drosophila genes, and provides access to hundreds of cancer-related TF-target subnetworks or “*regulons*”.

## Introduction

Precise regulation of gene expression is imperative for all biological processes. Sequence-specific transcription factors (TFs) bind to their DNA recognition sites within *cis*-regulatory elements and thereby contribute to the control of the transcriptional initiation rate of their target genes through an interplay with other transcription factors, co-factors, chromatin modifiers, and transcription factories [1]–[3]. The human genome encodes for about 1800 sequence-specific TFs, each of which regulates hundreds of target genes [1], [4], [5]. Because TFs play key roles in gene expression, they are often considered the master regulators of cellular processes. Thus, the mapping and characterization of their *regulon* (all the target genes of a TF) can provide crucial insight into the biological processes they control [6], [7]. For example, in cancer, ∼40% of the driver mutations affect TFs, and many of the key oncogenes and tumor suppressors, such as p53, MYC, E2F, and NF-κB, are transcription factors [8]. Identification of the TFs that operate a perturbed gene network, and detecting their target genes, are instrumental steps in uncovering key insights into oncogenic programs, including the discovery of therapeutic targets [9]–[12]. For example, although many target genes have been described for the tumor suppressor p53 [9], [13], [14], several aspects of the gene regulatory network (GRN) downstream of p53 remain unknown. For example, it is still unclear whether p53 also directly represses target genes; whether p53 cooperatively regulates target genes with particular co-factors; and whether different target genes are regulated depending on the cancer type, or depending on the context of p53 activation. The situation is obviously worse for less studied TFs for which often none or only few target genes are known.

The targets of a known TF can be identified experimentally with relatively high accuracy through chromatin immunoprecipitation followed by high-throughput sequencing (ChIP-Seq) [15]. However, ChIP-Seq has limitations because it is usually applied to cells in culture rather than to the actual biological sample (e.g., a tumor); and it focuses on a single TF at a time, that has to be chosen *a priori*. When the TF is not known in advance, or when only gene expression profiling can be performed, regulatory relationships can be uncovered by reverse-engineering a gene regulatory network starting from the expression data. One approach to solve this problem is by exploiting the fact that genes that are co-regulated by the same TF commonly share binding sites for this TF. However, detecting these short and variable TF binding sites (TFBS) within large non-coding regions represents a computational challenge when working with human or mouse genomes. Although a lot of progress has been made over the last decade and many motif discovery methods have been developed and refined (reviewed in [16]–[19]), motif discovery methods alone are not sufficient to map a gene regulatory network, nor can they be applied to noisy gene sets containing mixtures of targets of multiple TFs. This is true for both motif discovery methods relying on *de novo* detection and those relying on the enrichment of known position weight matrices (PWM). Additionally, many tools have a motif-oriented output, making it difficult to identify the possible upstream TF. A further limiting factor is that many methods are restricted to using human annotated PWMs (e.g. TRANSFAC [20], JASPAR [21] or UNIPROBE [22]), limiting the number of TFs that can be identified as candidate network regulators based on motif enrichment. Therefore, although *cis*-regulatory sequence analysis has great potential in resolving direct TF-target interactions, it has until today seen limited applications towards gene regulatory network mapping.

Finally, the recent availability of thousands of ChIP-Seq datasets, both from ENCODE [23], and other resources [24], yields new opportunities to discover master regulators from co-expressed gene sets [25], while at the same time pose challenges on how to integrate these data with motif discovery.

Here, we aim to tackle some of these challenges by increasing the performance of motif detection to yield high-confidence results, even in noisy gene sets. Motif detection is followed by the annotation of the discovered motifs with associated TFs and direct targets. To this end, we have collected more than nine thousand PWMs from various sources and from different species and link them to candidate binding TFs using a “motif2TF” procedure. This will allow the user to link hitherto anonymous motifs, and motifs of TFs from other species, to candidate human TFs. Furthermore we developed a user-friendly Cytoscape plugin [26], called iRegulon, allowing the integration of predicted *cis*-regulatory binding sites directly into a biological network. Finally, we extend and generalize this framework towards combined motif and track discovery on a co-expressed gene set, incorporating more than 1000 ChIP-Seq tracks. The iRegulon Cytoscape plugin is available via the Cytoscape App Store [27] and can be downloaded from http://iregulon.aertslab.org/.

## Results

### The iRegulon framework

**Figure 1:**
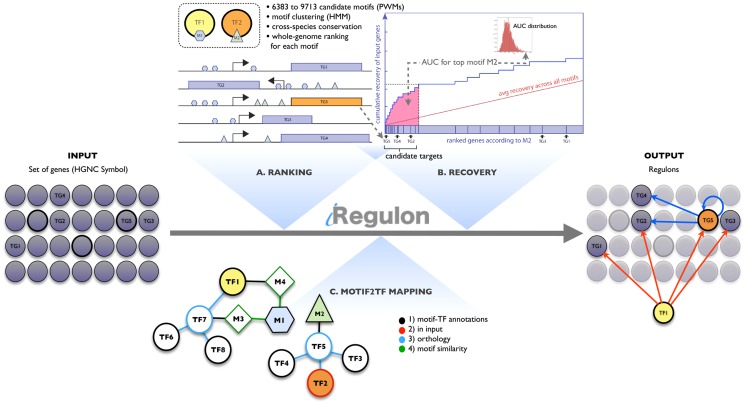
Regulon detection by rank-based motif discovery and motif2TF. Motif enrichment in iRegulon is measured using a *ranking-and-recovery* procedure using a large collection of position weight matrices (PWM). In the *ranking step* (**A**) all human genes are ranked for each motif by scoring for homotypic motif clusters across ten vertebrate species. In the *recovery step* (**B**) each of these gene rankings is tested against the set of input genes by calculating the Area Under the cumulative Recovery Curve (AUC, in pink). The example shown is for the top enriched motif, motif M2. The AUC score is normalized, based on the AUC scores of all motif rankings (distribution is shown as inset), to a normalized enrichment score (NES). A high NES score (≥3.0) indicates a motif that recovers a large proportion of the input genes within the top of its ranking. In parallel, the leading edge of the recovery curve is used to determine the optimal subset of genes that are likely controlled by this motif. In the last step (**C**) Motif2TF associates the candidate motif with (a number of) TFs by finding possible paths from a motif to a TF, in a motif-TF network based on direct evidence, orthology, and motif-motif similarity. The enriched TF can be from the input genes (e.g. TG5 encoding for TF2). See also Materials and Methods and Figures S1, S4.

The goal of iRegulon is to enable gene regulatory network mapping directly based on motif enrichment in a co-expressed gene set. As motif discovery method we have chosen to elaborate on the recent *ranking-and-recovery* methods [28]–[32] (Fig. 1). In the *ranking step* we generate whole-genome rankings of 22284 human RefSeq genes for a library of PWMs where a PWM is a matrix representation of a regulatory motif (Table 1). For each gene, a regulatory search space (500 bp, 10 kb or 20 kb around the Transcription Start Site (TSS), see Materials and Methods) is scanned for homotypic cis-regulatory modules (CRM) using a Hidden Markov Model [33] (Fig. S1). Starting from a library with *N* PWMs, *N* ranked lists of genes are generated, each with the most likely genomic targets of a particular motif at the top of the ranking [28], [29]. Next, orthologous search spaces in ten other vertebrate genomes are determined by UCSC *liftover* tool [34] and are subsequently scanned with the same PWMs. The rankings for different species are combined by rank aggregation [35] into one final ranking for each PWM in our library. For the PWM libraries we have collected and reformatted most of the available libraries into a “6K collection” (*N* = 6383 PWMs) and a “10K collection” (*N* = 9713 PWMs) (Table 1). These libraries contain PWMs from different species and also include candidate PWMs for unknown TFs. The results of the ranking step are *N* human gene rankings stored in an SQLite database. We also generated similar databases using mouse and *Drosophila* as reference species, in case the input gene set is derived from mouse or fruit fly.

**Table 1:** Description of the motif and track collections used.

Source	Organism(s)	Type of motif	# motifs “6K”	# motifs “10K”	# tracks “1K ChIP”
Elemento [73]	Drosophila	Predicted (conserved)^a^	371	371	-
FlyFactorSurvey [75]	Drosophila	B-1H, others (e.g., FlyReg)	614	652	-
hPDI [77]	Human	Experimental	437	437	-
Jaspar [21]	Multiple species	Curated	1315	1315	-
SelexConsensus [76]	Drosophila	Curated (FlyReg)	38	38	-
Stark [74]	Drosophila	Predicted (conserved)^a^	228	228	-
Tiffin [76]	Drosophila	Predicted (gene sets)^a^	120	120	-
TRANSFAC PUBLIC [5]	Multiple species	Curated, ChIP-chip	398	398	-
TRANSFAC PRO [5]	Multiple species	Curated, ChIP-chip	1153	1850	-
YetFasco [78]	Yeast	Uniprobe, Curated, ChIP-chip	1709	1709	-
ENCODE [79]	Human	Predicted (from DHS)^a^	-	683	-
Factorbook [46]	Human	ENCODE ChIP-Seq motifs	-	79	-
Taipale [132]	Human, Mouse	HT-Selex	-	820	-
iDMMPMM [133]	Human	footprints, Selex, b1h, peaks	-	39	-
SwissRegulon [134]	Human	Curated	-	190	-
Wolfe [135]	Drosophila	ZFP motifs	-	36	-
HOMER [116]	Multiple species	ChIP-Seq Motifs, others (e.g. ENCODE)	-	1865	-
Dimers [136]	Human	Predicted dimers	-	603	-
ENCODE ChIP-Seq [23]	Human	-	-	-	999
Taipale ChIP-Seq [24]	Human	-	-	-	117
p53 and control ChIP-Seq (this study)	Human	-	-	-	2
**Total**			**6383**	11611 (**9713** nr)	**1118**

a Orphan motifs (unknown TFs).

nr = non-redundant.

The *recovery step* uses as input any set of co-expressed genes (Fig. 1B). The enrichment of these genes is determined in each of the *N* motif-based rankings using the Area Under the cumulative Recovery Curve (AUC), whereby the AUC is computed in the top of the ranking (default set to 3%, see Fig. S2 for validation). The AUC values are normalized into a Normalized Enrichment Score (NES) on which we set a default cutoff of 3.0, corresponding to a False Discovery Rate (FDR) between 3% and 9% (Fig. S3 and Materials and Methods). The leading edge of candidate targets is selected as the optimal subset of highly ranked genes compared to the genomic background and compared to the entire motif collection as background (Fig. 1B and Materials and Methods).

We have previously successfully applied the *ranking-and-recovery* method for *Drosophila*, namely in cisTargetX [29] and i-cisTarget [28]. These methods have been proven successful in identifying upstream regulators and direct target genes from co-expressed gene sets for Atonal [29], Shavenbaby [36], Fruitless [37], EcR [38], Dichaete [39], Glass [40], dJun/Vri [41], and Rfx [42]. Here, we apply this framework for the first time to human and mouse and we add two novelties to facilitate GRN mapping. The first is a *motif2TF* procedure that links an enriched motif (PWM) to a candidate binding TF (Fig. 1C and Materials and Methods). For this step we constructed a database of motif-TF direct annotations, TF-TF edges as defined by gene homology [43], [44], and motif-motif edges as defined by motif similarity (using Tomtom [45]). The database links 6031 motifs from the “10K” collection to 1191 human TFs. The advantage of this method is that it allows discovery of motif-TF links based on orthology and based on similarities between annotated and “unknown” motifs in the collection. Application of this method adds 247 more TFs to be identified than the 944 directly annotated TFs in human, and vastly increases the number of different motifs per TF (see Materials and Methods for more detailed description). The second novelty is the availability of the method as a Cytoscape [26] plugin, called *iRegulon*. The plugin works on any input network and returns a combination of regulators, their direct targets within the input network, and their binding motifs. A detailed description on the use of the plugin is provided in Fig. S4. This is, to our knowledge, the first method that brings *cis*-regulatory sequence analysis into Cytoscape. This dramatically changes the way motif discovery is performed, because instead of a list of promoter sequences used as input, now any set, network, or pathway of genes can be used as input. Instead of a list of enriched motifs, *regulons*, are the output, containing the candidate TFs along with their optimal direct target subsets. iRegulon results can be immediately used to map (or annotate) gene regulatory networks and be integrated with the extensive array of regulatory, expression, and annotation tools available within Cytoscape.

**Figure 2:**
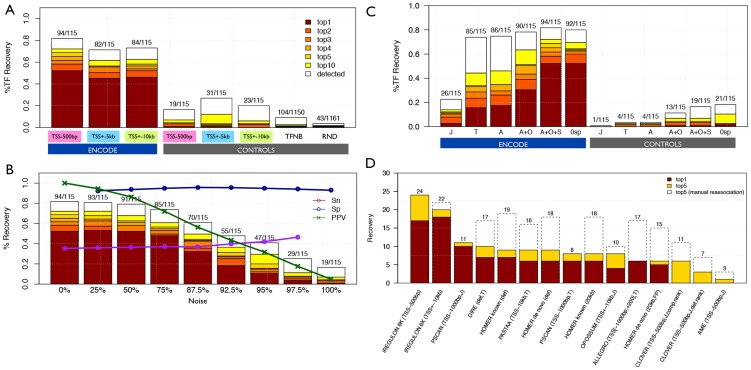
Evaluation of iRegulon and comparison to other methods. The TF recovery (y-axis) corresponds to the fraction of TFs correctly detected among all TFs for which a motif from our library can be associated. **A.** Positive sets consist of the top 200 genes ranked by the maximum signal value of the ChIP-Seq peak in the corresponding search space. Control sets are negatives from ENCODE (genes without a ChIP-Seq peak); TF neighborhoods (TFNB; all TFs within 5 Mb around a query TF); and random signatures (RND). The color (from red to yellow) and order of stacked bars indicate the number of times the queried TF was identified in the 1^st^ rank (top1), 2^nd^ rank (top2), 3^rd^ rank (top3), 4^th^ rank (top4), 5^th^ rank (top5) and 6^th^ to 10^th^ rank (top10). White color indicates the number of detected TFs (motif enrichment ≥3) but with rank >10. **B.** Positives are mixed with negative genes (noise) from 0% to 100% of noise. The lines represent the sensitivity (Sn), Specificity (Sp), and Precision or Positive Predictive Value (PPV) of target gene selection. **C.** The layers of motif2TF increase the performance. Recovery for ENCODE signatures and their control sets using different motif2TF parameters: 1) Motif collection effect (J, T, A barcharts), 2) Homology effect using a threshold on Identity% for all motifs (A+O barcharts), 3) Motif similarity effect using a threshold on the p-value (A+S barcharts), and combinations (A+O+S). Only Jaspar motifs (J); Only Transfac Pro (T); All motifs from Jaspar and Transfac pro, and others databases (A); All motifs+Orthology (A+O); and All motifs+Orthology+Similarity (A+O+S); blue indicates the analysis done on ENCODE sets and grey indicates on the control sets. **D.** Tool comparison using a benchmark of 30 gene sets constructed as the top 200 target genes based on ChIP peak occurrences in the 20 kb regulatory region for 30 TFs (these TFs were selected from FactorBook having their canonical motif as top enriched in the actual ChIP peaks). The number of times the queried TF was identified in the top1 (red) and top5 (yellow) is recorded. The dashed boxes represent top 5 recoveries if similar motifs are manually re-associated to the query TF. Default parameters were used, but when possible, they were adjusted to use the tss-centered-20 kb regions. See also Figures S2–S3 and Table S1.

To evaluate the performance of iRegulon, we derived direct target gene sets for 115 sequence-specific TFs from the ENCODE ChIP-Seq data [46], and for each target set we investigate whether the ChIP'ped TF can be correctly recovered (see Materials and Methods). Out of 115 tested TFs, iRegulon correctly identifies up to 94 TFs (82.6%) with Normalized Enrichment Scores (NES) above 3 (Fig. 2A, and Materials and Methods). We found iRegulon to be robust to noisy gene sets by adding increasing levels of noise (negative genes) to each set of targets (Fig. 2B). The *motif2TF* step is crucial to link an enriched motif to a candidate TF; and including motifs from other species and unknown motifs allow detecting many more correct regulators compared to using only known human motifs from TRANSFAC or JASPAR (Fig. 2C). After optimizing the parameters of iRegulon and *motif2TF* (see Materials and Methods and Fig. S2), we compared iRegulon with eight other motif discovery methods that use a similar input (a set of co-expressed genes) and generate a similar output (candidate regulators) using a non-ambiguous subset from Factorbook [46] (Materials and Methods). iRegulon identifies the correct TF at the first position in 17/30 cases while the other tools on average detect only 5.1/30 TFs at the first position (Fig. 2D, Table S1). Interestingly, the improved performances of iRegulon are not only due to the large PWM collection and the *motif2TF* mapping. Indeed, iRegulon still outperforms the other methods when using only the JASPAR collection and disabling the *motif2TF* step (Fig. S2C) or *vice versa*, when manually promoting similar motifs in the other tools to the correct TF (dashed bars in Fig. 2D). As expected, the true positive target gene recovery is significantly higher when iRegulon uses a 20 kb search space around TSS compared to using only the proximal promoter (Wilcoxon rank-sum paired test, p-value = 0.004) (Fig. S2D). We conclude that the core motif discovery framework of iRegulon is better than other tools, and that the large motif collection and the *motif2TF* step deliver a marked step forward in TF identification performance.

### Regulons can be discovered from various types of noisy and heterogeneous gene sets

In the validation and benchmark analyses above we used gene sets derived from ENCODE ChIP-Seq data as input for iRegulon. In this section, we explore more realistic types of inputs, such as co-expressed genes downstream of a TF perturbation [47]; genes involved in the same signaling pathway (e.g., KEGG [48], Reactome [49] or Gene Ontology [50]); highly connected genes in a biological network (e.g., GeneMania [51] or STRING [52]); shared targets of a common microRNA. In the first example, we applied iRegulon to a set of 171 genes that are significantly up-regulated under hypoxia [53]. iRegulon yields a top-scoring regulon that contains HIF1A as master regulator, along with 94 predicted direct target genes (Fig. S5A). The predicted HIF1A targets are likely functional targets because they overlap much more (41%) with known HIF1A targets [54] than the non predicted targets (15%). More systematically, when applied to 76 co-expressed gene sets obtained after a genetic perturbation of the TF (gene sets from MSigDB [47]), the perturbed TF is recovered in 38 cases (50%) and as the top ranked master regulator in 18 cases (24%). The lower recall to detect the correct upstream TF compared to ChIP-derived gene sets is expected because not all TF perturbation experiments successfully result in significant gene expression changes of the direct target genes.

Next, we analyzed a set of 161 genes involved in the NOTCH signaling pathway and identified the top two regulons to be controlled by *HEY1/HEY2/HES1* and *RBPJ*, two major players involved in NOTCH signaling (Fig. S5B). We also analyzed 1198 genes involved in immune response (GO:0006955), and as expected we found the *IRF* and REL/NF-κB regulons, with 806 and 711 direct target genes respectively, highlighting their role as master regulators of the immune response (Fig. S5C). We also analyzed all 2233 TF-centered subnetworks within protein association networks and found enrichment of direct targets for 151 (13.2%) and 159 TFs (14.6%) for GeneMania and STRING networks, respectively, indicating that transcriptional interactions are partially represented in protein-protein interaction networks as well (Fig. S5D). Finally, we analyzed 159 sets of known microRNA targets, for which iRegulon identified significant cross-talks (feed-forward loops) between the predicted TF and microRNA regulons (Fig. S5E). While previous methods have thus far been validated and applied to co-expressed gene sets derived from gene expression profiling, here we show that motif discovery with iRegulon can quickly identify master regulons on diverse types of gene sets, as long as a small fraction of the input set is directly co-regulated by the same TF.

### Mapping a gene regulatory network downstream of p53

We now applied iRegulon to study the gene regulatory network downstream of the p53 tumor suppressor. p53 functions mainly, if not exclusively, as a TF which regulates the expression of hundreds of genes that in turn mediate its biological activities including induction of cell-cycle arrest, senescence and apoptosis [55], [56]. Although p53 is one of the most-studied transcription factor and hundreds of target genes have already been identified [14], [55], many aspects of its downstream network remain unresolved and a more comprehensive understanding of the p53 downstream signaling network is crucial given its importance in oncogenesis.

**Figure 3:**
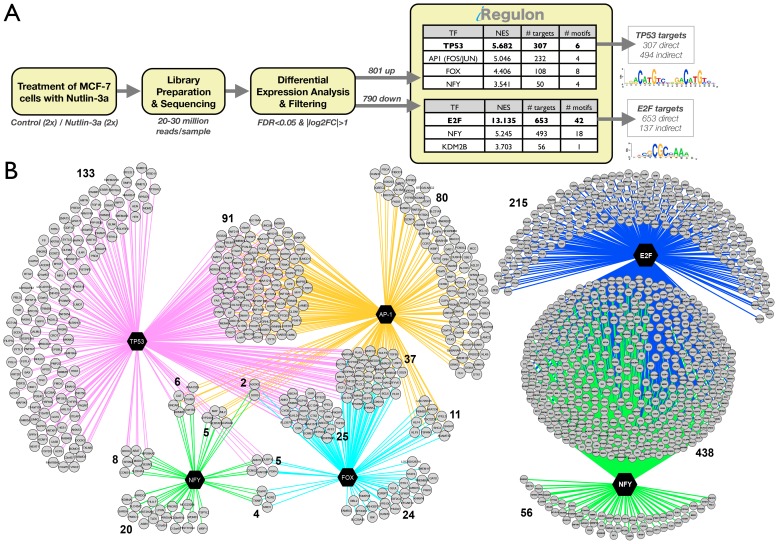
Using iRegulon to map a p53-dependent gene regulatory network. **A.** MCF-7 breast cancer cells were treated with Nutlin-3a to stabilize p53, followed by RNA-Seq after 24 h. iRegulon results shows p53 as top regulator in a set of 801 up-regulated genes, represented by 6 significantly enriched motifs, and 307 predicted direct targets. The top regulator in the set of down-regulated genes is E2F, with 653/790 predicted direct targets. **B.** Regulatory network for up-regulated target genes showing the overlap between the p53 regulon and regulons of predicted co-factors (AP-1, NFY, FOX) and regulatory network for down-regulated target genes showing a strong overlap between the predicted E2F and NF-Y regulons. Targets are in grey circle nodes and TF in black hexagon nodes. Regulons for each TF are represented by different edge colours. See also Tables S2–S5.

We first determined a p53-dependent gene signature in the MCF-7 human breast cancer cell line by RNA-seq upon stabilization of p53 by the non-genotoxic small molecule Nutlin-3a [57]. This treatment resulted in significant up-regulation of 801 genes and down-regulation of 790 genes. Both up- and down-regulated gene sets were subsequently analyzed with iRegulon (Fig. 3A). The top-scoring regulon in the list of up-regulated genes is confirmed as the p53 regulon, with 307 genes predicted to be direct targets (Fig. 3A and Table S2). This indicates that p53 itself is the master regulator of the downstream network and directly controls many up-regulated genes, but not all of them (at least 38%). A Gene Ontology (GO) enrichment analysis of the 307 predicted direct targets identifies p53-related processes and pathways, such as “p53 signaling pathway” (adjusted pvalue = 3.18e-21) or “Apoptosis” (adjusted p-value = 6.76e-07), while the set with the remaining 494 up-regulated genes show no significant GO term enrichment (data not shown).

In this particular experimental setup the master regulator, namely p53, was specifically perturbed and thus known *a priori*. Yet, even under such circumstances there are two important advantages of using a computational regulatory analysis with iRegulon. First, the explicit finding of the p53 motif as top ranked indicates that p53 *directly* controls a large portion of the up-regulated genes but not all, creating two clearly distinct subsets. Second, we discover potential p53 co-factors and secondary regulons downstream of p53. Particularly, among the 801 genes that are activated downstream of p53, we found three other regulons, one operated by activator protein 1 (AP-1, heterodimer composed of JUN/FOS/FOSL1/FOSL2), another by a Forkhead TF (FOX), and another by NF-Y (Fig. 3A, Table S3A). These secondary regulons show extensive overlap with the primary p53 regulon, indicating that these TFs may be important contributors in gene regulation downstream of p53 (Fig. 3B). The AP-1 regulon, sharing 136 genes (59% of its regulon) with the p53 regulon might indicate a prevalent co-factorship between the two proteins, something that has been reported before but never on such an extended scale [58], [59]. In addition, one of the shared p53-AP1 targets is *GADD45A*, a gene involved in DNA damage repair, that has been shown to be a *bona fide* target of both p53 and AP-1 [60]. Interestingly, two subcomponents of the AP-1 complex, *FOS* and *FOSL1*, are themselves up-regulated upon p53 stabilization, and are among the predicted direct p53 targets (Table S4). These results, together with the fact that the AP-1 motif was not enriched among the down-regulated genes indicate a positive, synergistic effect of the p53 and AP-1 regulons.

Nutlin-3a treatment also resulted in 790 significantly down-regulated genes. Interestingly, the analysis of this set with iRegulon does not detect the p53 motif as enriched. It does however identify E2F as master regulator with an astounding 653 (82.7%) predicted direct targets (Table S3B). Moreover, three E2F family members, namely E2F1, E2F2, and E2F8 are all strongly and significantly down-regulated upon Nutlin-3a treatment (around 10-fold down with p-value<1.0E-64), indicating the marked involvement of this protein family in the repressive mechanisms of p53. Similarly, iRegulon points towards NF-Y as an important second master regulator of a large number of down-regulated genes (493 genes). Both E2F and NF-Y have been reported as important players for p53-mediated down-regulation of genes [61], [62]. This may happen through p21 regulated cyclin dependent kinases, resulting in a lack of phosphorylation of NF-Y and Rb which ultimately renders both NF-Y and E2F (through Rb) inactive [63], [64]. Interestingly, the majority of NF-Y's predicted regulon overlaps with that of E2F, with only a very small number of genes predicted as NF-Y only targets (Fig. 3B). The enriched Gene Ontology terms of these overlapping target genes are related to cell-cycle processes, an expected result since both E2F and NF-Y have been established to regulated cell cycle-related genes, often in a cooperative manner [65]–[67]. In contrast to E2F, NF-Y itself is not down-regulated as a gene by p53 activation. However, it is possible that NF-Y is regulated at the protein level rather than at the transcriptional level in response to p53 activation. All together, these findings support the notion of an indirect rather than a direct p53 repressive process largely working through the p53-p21 axis, which affects both E2F and NF-Y [63], [68]. All together, iRegulon generates marked ideas concerning p53, which are further elaborated upon in the next section.

### ChIP-Seq on p53 and E2F confirm their predicted regulons

**Figure 4:**
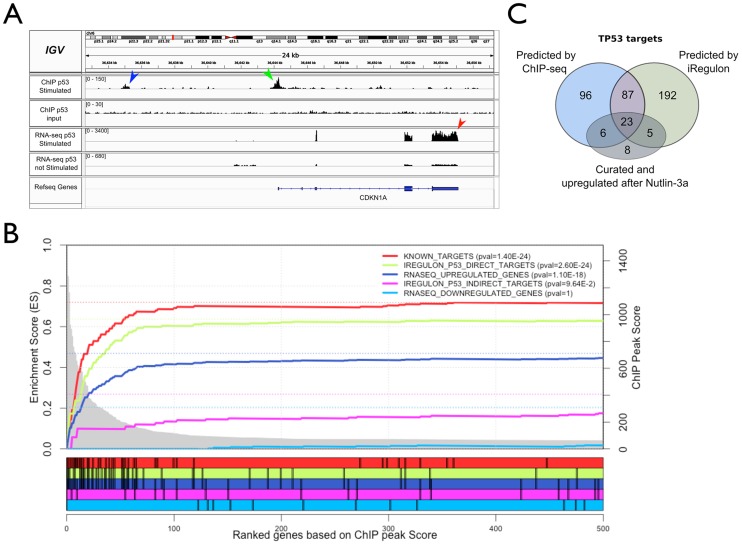
Validation of the p53 regulon by ChIP-Seq. **A.** Integrative Genomic Viewer (IGV) [131] screenshot for *CDKN1A*, a known p53 target gene, showing up-regulation by RNA-seq (red arrowhead) and ChIP peaks in the upstream region (green and blue arrowhead). IGV is free software under GNU Lesser General Public License, version 2.1 (LGPL-2.1). **B.** Gene Set Enrichment analysis, with on the x-axis all genes in the genome ranked according to their maximum ChIP-Seq peak (20 kb around TSS). The p53 targets (green curve) show higher enrichment than the total set of up-regulated genes (blue curve), approaching the previously known curated targets (red curve), while the non-predicted p53 targets (magenta curve) and the set of down-regulated genes (cyan curve) show no enrichment. The initial two steps in the magenta curve represent two false negative predictions of iRegulon (they fall just below the optimal cutoff), namely *PLK3* and *DDB2*, which are up-regulated and have a ChIP peak. P-values in the legend are calculated by the hypergeometric formula of the leading edge determined by GSEA. **C.** Comparison between annotated up-regulated p53 targets and predicted p53 targets by iRegulon and ChIP-Seq, indicating the number of previously known p53 targets. See also Figure S6.

To test the predicted p53 regulon we determined the genome-wide chromatin occupancy by p53 in Nutlin-3a stimulated MCF-7 cells using high-coverage ChIP-Seq (∼30 Million uniquely mapped reads). Fig. 4A shows the raw ChIP-Seq data for the known p53 target *CDKN1A*, with a very strong peak overlapping the known p53 binding site in the promoter of *CDKN1A* [69]. To avoid arbitrary thresholds on peak calling we used lenient peak calling settings to rank all genes in the genome according to their likelihood of being a p53 target based on ChIP peaks only (see Materials and Methods). To assess whether this ranking yields true p53 targets on top, we curated 223 *bona fide* p53 targets from the literature and public databases (Table S5), and indeed found these targets to be significantly enriched in the top of this ranking (Fig. 4B, p-value = 1.40E-24). Within the same ranking, the 307 predicted p53 targets by iRegulon are nearly as significantly enriched in the top as the curated targets (p-value = 2.60E-24), while the 494 remaining up-regulated genes are not significantly correlated with the ChIP peak data (p-value = 0.096). Importantly, this result shows that iRegulon is not only able to identify the master regulator, but is also able to correctly distinguish between direct and indirect targets from a set of co-expressed genes. Only two up-regulated genes with a high ChIP peak, namely *PLK3* and *DDB2*, were missed by iRegulon. About 100 up-regulated genes have a small ChIP peak but have not been predicted by iRegulon as target genes. These peaks are likely false positive ChIP peaks because they do not show p53 motif enrichment when analyzed separately (Fig. S6A–C). Finally, to compare how many targets are missed by iRegulon, and how many by ChIP-Seq, we again used the set of curated targets, and found comparable numbers of false negatives, namely six for iRegulon and five for ChIP-Seq (Fig. 4C). In the previous section we had also found that gene repression downstream of p53 is indirect through E2F, which has been shown recently to be mediated by p21 and RB [63], [68]. If this is true, then the down-regulated genes should not contain p53 ChIP peaks. To test this, we plotted the recovery of the 790 down-regulated genes along the p53 ChIP-peak-based gene ranking generated above (Fig. 4B). Similar to the indirect up-regulated genes, the down-regulated genes are completely depleted of p53 ChIP peaks (p-value = 1.0). On the other hand, the down-regulated genes are positively correlated with E2F1 ChIP-Seq data in MCF-7 from ENCODE (Fig. S6D). When combining all the small p53 ChIP-Seq peaks that are detected amongst the down-regulated genes, the p53 motif is not found by *de novo* motif discovery, while the ChIP peaks of direct up-regulated targets are strongly enriched for *de novo* p53 motifs (Fig. S6A–C). From the ChIP-Seq validation data, we conclude that iRegulon predicts the correct master regulators (p53 and E2F) and that predicted target genes of these TFs significantly overlap with ChIP-Seq derived targets. By combining iRegulon and ChIP-Seq data, we propose a set of 110 “top targets” of p53 in MCF-7 that are directly and positively regulated. When further comparing these predicted targets to recent reports of several p53 targetomes based on combining gene expression profiles with p53 ChIP-Seq data under different experimental conditions [58], [59], [68], we could confirm many common targets, but also uncovered 56 new direct p53 target genes with our analysis (Table S6).

### New p53 targets are confirmed by meta-analysis across human cancers and by enhancer-reporter assays

**Figure 5:**
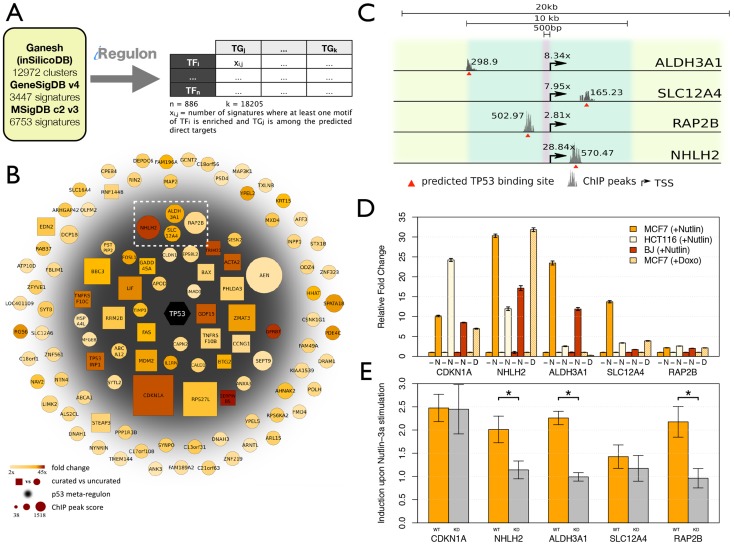
Validation of p53 target genes and target CRMs. **A.** Workflow to generate meta-regulons. Meta-regulons can be obtained directly via the iRegulon Cytoscape plugin. **B.** Direct targets of p53 in MCF-7 cells. All genes are significantly up-regulated by p53, are predicted as p53 targets by motif discovery in iRegulon and have a significant ChIP peak. In addition, genes in the grey shaded inner circle are part of the p53 *meta-regulon*, meaning that they are also found as p53 targets across cancer signatures. **C.** Four new p53 target genes are presented in detail. **D.** Relative mRNA expression levels of p53 target genes before (−) and 24 h after stimulation with 10 µM Nutlin-3a (N) or after 1 hour pulse of 5 µM Doxorubicin (D). Expression is shown relative to non-treated control and normalized to optimal reference genes for each cell type, assessed by GeNorm [130]. Error bars show standard error of the mean (SEM) of 3 replicates. **E.** Enhancer-reporter assays of four predicted p53 target CRMs, after transfection into MCF-7 cells before and after induction with Nutlin-3a (5 µM) in Wild Type and p53 Knock-down MCF-7 cells. Error bars represent SEM of 5 replicates. See also Figures S7–S8 and Tables S4,S6.

To explore the relevance of the newly identified p53 targets in other tumor types, we applied iRegulon in a meta-analysis to about twenty thousand cancer gene signatures, i.e. differentially expressed genes obtained from cancer specific experiments. We reasoned that those target genes that are recurrently predicted across cancer gene signatures, might contribute to the tumor suppressor role of p53. We used gene signatures from GeneSigDB [70], MSigDB [71] and from gene modules generated across 91 large cancer microarray data sets (see Materials and Methods and Fig. 5A). Out of 23172 signatures, p53 is found as regulator in 709 signatures. We merged the direct p53 targets across all these signatures into a network and weighted the edges according to the recurrence of this p53-target interaction across all signatures. Many previously known p53 targets and many ChIP-Seq derived targets are recovered using this analysis (GSEA NES = 3.01, FDR<0.001) (Fig. S7). Of the 110 predicted p53 targets in MCF-7 cells (as defined above), 44 are also predicted as p53 target in cancer gene signatures (grey area in Fig. 5B). These genes are predicted as p53 targets by iRegulon *and* show a significant ChIP peak *and* are represented in the p53 cancer-related meta-regulon. Amongst these 44 genes, 20 were previously indicated as well established p53 targets (genes in squares in Fig. 5B). When extending the analysis and including target genes recently reported in literature [58], [59], [68], it becomes clear that most overlap coincides within this metatargetome (34/44) (Table S6). Keeping in mind that many of the p53 targets reported by others were found using different cell lines, the enriched overlap within this metatargetome can be interpreted as a sign that these genes represent a core set targeted by p53 regardless of the cell type. Interestingly, when looking at targets like *RAP2B*, *NHLH2*, *SLC12A4*, and *ALDH3A1*, they could not have been identified through motif discovery in proximal promoters only, because the p53 binding sites are located either further upstream (∼1 kb for *RAP2B* and ∼5 kb for *ALDH3A1*) or in introns (*NHLH2* and *SLC12A4*) (Fig. 5C).

Next we confirmed experimentally whether these four targets are *bona fide* p53 transcriptional targets. They are all induced in a p53-dependent manner in various cellular model systems including normal diploid human fibroblasts (BJ cells) and various cancer cell lines (i.e. HCT116 and MCF-7) (Fig. 5D). Except *ALDH3A1*, they are also all significantly induced upon exposure to the DNA damaging agent doxorubicin, a well-established p53 inducer (adjusted p-value<0.05). Their kinetic of induction both in response to Nutlin-3a and DNA damage is comparable to the one seen with known direct p53 targets such as *CDKN1A* further supporting a direct role for p53 in their regulation (Fig. S8). Finally, for all except one we could confirm luciferase reporter activity of the predicted p53 enhancer region (Fig. 5E). Enhancer-reporters for *ALDH3A1*, *NHLH2* and *RAP2B* show a significant induction after Nutlin-3a treatment in wild type but not in a p53 knock-down (KD) cell line (p-value<0.05). *SLC12A4* does not have a significant induction in either cell-type. Note that our positive control enhancer, namely the *CDKN1A* promoter, is a very responsive p53 target and likely responds to low levels of p53, which could explain the induction that is still observed even under p53 KD conditions. Functionally, these validated p53 target genes have been implicated in p53-regulated processes such as the control of cell volume, growth and movement (SLC12A4 and RAP2B) and metabolism (ALDH3A1 and NHLH2).

### Motif and track discovery join forces

**Figure 6:**
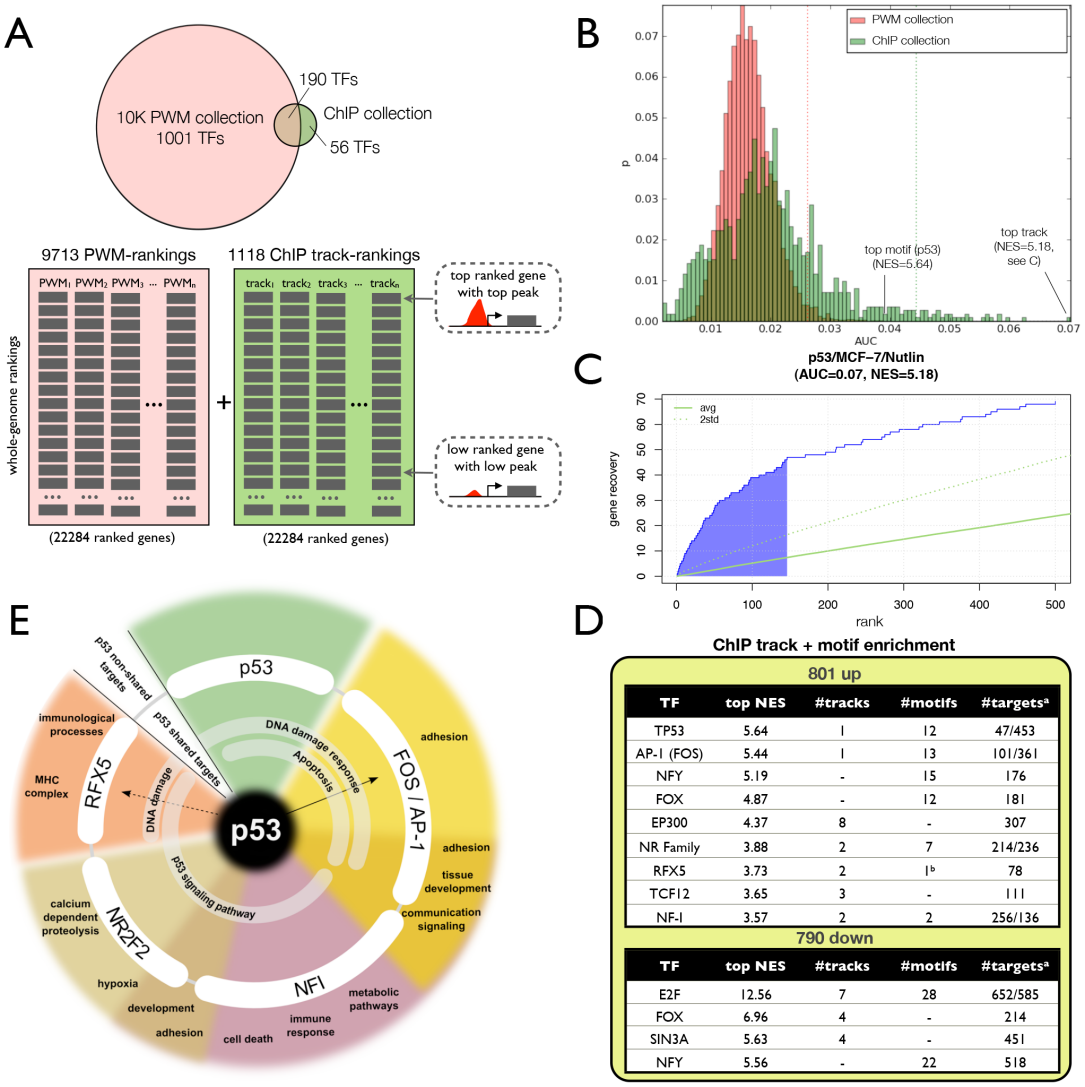
Combined analysis using 10K motifs and 1K ChIP-Seq tracks. **A.** Two ranking databases were made using 9713 motifs and 1118 ChIP-Seq tracks. The ChIP-Seq tracks consisted of all ENCODE and Taipale ChIP-Seq data against TFs, and the p53 ChIP-Seq track generated in this study. **B.** AUC distributions for ChIP-Seq and motif rankings, using the p53 signature as input. **C.** The actual recovery curve for the p53 motif and track. Shaded area indicates the AUC. **D.** Top enriched ChIP tracks and motifs on the up- and down-regulated gene sets (NES>3, except for RFX5 motif that was detected with NES = 2.82 (b). (a) Predicted targets are shown for both enriched tracks and motifs respectively. **E.** Functional categories found enriched for predicted co-factors of p53. The annotation of p53-shared targets is shown in the inner circle, while the annotation of non-shared targets (for example, AP-1 targets but not p53) is shown on the outer circle. The co-factors shown here are those found by both motif and track enrichment (see also Table S7).

We extended our *motif discovery* approach to allow the discovery of significantly enriched ChIP-Seq tracks in a set of co-expressed genes. We created a database with track-based gene rankings from a collection of 1118 ChIP-Seq experiments against 246 human sequence-specific TFs across 40 cell types and apply the same “ranking-and-recovery” enrichment calculation as employed earlier (see Materials and Methods). These and other recent resources further enlarged our motif collection to 9713 distinct PWMs (“10K collection”) (Table 1). To test whether motif and track discovery can be performed simultaneously, we combined the motif-based rankings and the track-based rankings into one enrichment analysis, although each AUC score distribution is kept separate for normalization (Fig. 6A–B). Applied to the 801 p53-dependent up-regulated gene set, the combined approach still detects p53, AP-1, NFY, and FOX in the top motifs. Both for p53 and AP-1, enriched ChIP-Seq tracks are found by the track discovery, being our in-house performed p53 ChIP-Seq in MCF-7 after Nutlin-3a (ranked first of all tracks, NES = 5.18) and the FOSL2 ChIP-Seq tracks in MCF-7 from ENCODE (NES = 3.30) (Fig. 6C–D, Table S7). In addition, we found five more candidate TFs with a putative role in the network downstream of p53 that were not detectable using the 6K motif collection only (Fig. 3). Three of these additional candidates, namely RFX5, NR2F2, and NFI have both their ChIP-seq track and motif enriched while two more candidates, namely p300 and TCF12 only show track enrichment (Fig. 6D). To our knowledge, no interaction of these TFs with p53 has been reported in the literature. Although the targetomes of the co-factors overlap to some extent (20–42%) with p53 targets, they have a considerably large set of target genes independent of p53. Hence, with these additional TFs added downstream of p53, we can once more explain an additional fraction of the up-regulated gene set, with all the ChIP-Seq track-derived interactions together regulating 542 of the 801 genes. RFX5 is of particular interest since the gene itself is strongly up-regulated by p53 and is in fact among the core set of 801 up-regulated genes (log2FC = 1.9 and adjusted p-value = 1.05E-15). RFX5 is mainly known as a regulator of MHC-II genes, and indeed, among the top predicted RFX5 target genes downstream of p53 we find HLA-F, MR1, and other genes involved in antigen and interferon-related processes. Interestingly, RFX5 has recently also been shown to act as a DNA mismatch repair stimulatory factor [72], and several p53-shared RFX5 targets, such as DDB2 and BBC3, are in fact related to DNA damage response (adjusted p-value = 6.99E-5, Wikipathway ID:WP707) (Fig. 6E). Hence, RFX5 can be considered as a new candidate co-factor to modulate certain aspects of the p53-regulated response, and may explain why MHC-II genes are up-regulated in a p53-dependent manner. This proof-of-principle of combined motif and track enrichment paves the way towards further integration of regulatory track data and enhancer prediction data to map gene regulatory networks.

## Discussion

We have optimized and expanded motif discovery methods and used large collections of up to 10.000 candidate motifs to facilitate translation of motif detection results into a network biology framework. By adding this network-layer on top of *cis*-regulatory motifs, we could generate direct insight into a biological process, rather than producing a mere list of enriched motifs from a gene set. iRegulon outperformed existing methods at detecting the correct upstream regulator. We found that using PWMs from other species than human greatly helps motif detection in human data sets. Many TFs are conserved from human to mouse, and even from human to fly or yeast, and sometimes the yeast or fly PWM is of higher quality or better captures the specificity of DNA binding. In addition, we found that using multiple PWMs for the same TF is an advantage and leads to higher performance of TF recovery compared to using non-redundant motif collections. Our motif collection also contains an important fraction of “novel” motifs for unknown TFs. These motifs are mostly derived from whole-genome computational predictions. In some cases these unknown motifs are clustered together in the output of iRegulon alongside a known motif, and can thereby lead to candidate TF predictions, while in other cases they may represent orphan motifs (with unidentified TFs). The mixture of known and unknown motifs creates a hybrid motif detection approach, combining *de novo* motif discovery and pattern matching approaches.

Large-scale analyses of co-expressed gene sets of different origins, including co-expression, TF binding (ChIP), protein-protein association networks and microRNA targets, suggest that by exploiting the genome sequence, together with other species' genomes and collections of consensus TF binding sites, the most relevant sub-networks that underlie observed changes in gene expression or observed genetic interactions can be reconstructed. In up to 70% of the cases, the upstream regulatory factor can be identified, along with a set of direct targets. Therefore iRegulon provides an alternative approach to probe a particular biological process when gene expression data is available but the TF is not known in advance and/or ChIP-Seq is not feasible. By combining iRegulon with RNA-Seq, the resolution of gene expression profiling and gene regulatory network mapping can be increased, allowing the characterization of any cell type, cellular response, or tumor sample, up to the single cell level.

Multiple regulons are often discovered from one co-regulated gene set. This is expected because in higher vertebrates gene regulation is combinatorial, where multiple TFs cooperate, either through binding in the same CRM (called heterotypic CRMs), or in separate CRMs of the same target gene [17]. In addition, the targets of a TF can be TFs themselves, and in turn activate or repress their own targets. For example, in the p53-dependent gene set iRegulon identified not only p53 as regulator, but also a previously known co-factor AP-1 and new regulators downstream of p53 such as RFX5. Interestingly, *FOS* and *FOSL1*, important members of the AP-1 complex, and *RFX5*, were all identified in this study as targets of p53. These regulators can explain a large proportion of the possible target genes of p53 as being indirect and regulated by another TF. When we extended our *ranking-and-recovery* framework to include more than one thousand ChIP-Seq data tracks, we also found the respective ChIP-Seq peaks for AP-1, RFX5, and several other co-factors as significantly enriched in the p53 downstream network. The joint finding of both a motif and a track for the same transcription factor strongly increases the confidence for these factors to play a role in the network as master regulator (i.e., directly controlling many target genes). Nevertheless, we envision that in most cases the motif enrichment alone, without any track enrichment, can directly lead to candidate master regulators, because ChIP-Seq data is condition-specific and is currently available for relatively few transcription factors.

The absence of a regulator in the output of iRegulon, when neither a motif nor a track is enriched, can also be informative. For instance, neither the p53 motif nor its ChIP-Seq track are found enriched among the down-regulated genes, leading to the hypothesis that p53 does not act as a direct repressor, but only as an activator. Rather, iRegulon points to E2F as the master regulator of the down-regulated genes, both by its motif and track. This finding can be explained as indirect down-regulation of E2F targets and has recently been experimentally established: p21 controls RB1-mediated repression of E2F targets, including E2F family members themselves, thereby reinforcing this signal further [63], [68].

Our experimental findings on the p53 regulon were obtained in MCF-7 breast cancer cells. Usually, one iRegulon analysis is focused on one biological process, and predicts transcriptional targets that are relevant in that particular cell type or condition under study. We show that it is also possible to apply iRegulon more systematically on multiple signatures to identify cancer-related ‘meta-regulons’. They often represent the canonical, high-confidence target genes and agree well with ENCODE ChIP-Seq data (Fig. S7). This shows that relevant TF-target interactions can be identified purely from the genome sequence, thereby creating a valuable resource for less studied TFs.

## Materials and Methods

### Sequence search spaces

Three predefined regulatory search spaces are used in this manuscript from small to large regions: 500 bp upstream of TSS [TSS−500 bp,TSS]; 10 kb around TSS [TSS−5 kb,TSS+5 kb]; 20 kb around TSS [TSS−10 kb,TSS+10 kb]. If another gene is located within the upstream region, then the region is cut where this neighboring gene begins or ends (depending on which strand this gene is located on). Coding exons are excluded from the search space to avoid bias towards these exons through conservation. Notice that there can be multiple regions per gene (various upstream regions for alternative transcripts, and multiple introns) (see example in Fig. S1). When multiple regions are scored for a given gene, the rank of the highest ranked region is taken into account as the final rank of the gene.

### PWM-based whole-genome rankings across species

Motif detection relies on an offline scoring step whereby every gene in the human genome, along with orthologous sequences in ten other vertebrate genomes, is scanned with *Cluster-Buster* [33] for *homotypic clusters* of motifs using a library of *N* position weight matrices (PWMs), generating a database of *N* ranked lists of genes, each with the most likely genomic targets of a motif at the top of the ranking.

#### 1) Motif collection

The library of motifs used in this manuscript is comprised of 6383 PWMs from several sources [73], [74] and databases: TRANSFAC [5], Jaspar [21], FlyFactorSurvey [75], SelexConsensus [76], hPDI [77], YeTFaSCo [78] and Tiffin [76] (Table 1). The motifs are collected as count matrices (scaled to 100 when the source matrix is a position-frequency matrix). Redundant PWMs (i.e. exactly the same count matrices annotated independently by different sources) are discarded. Importantly, note that we didn't use the motifs derived from ENCODE ChIP-Seq data that are published recently (76 from Factorbook [46] and 683 motifs in ENCODE [79]) to avoid over-fitting in our *in silico* validation. This motif collection (excluding TRANSFAC PRO motifs) is publicly available from http://iregulon.aertslab.org.

#### 2) Conservation information

Each gene is identified by its HUGO Gene Nomenclature Committee (HGNC) identifier and the whole-genome ranking for human (hg19) is comprised of 22284 genes. The LiftOver utility from the UCSC Genome Browser [34] was used to obtain orthologous regions between different vertebrate genomes. Vertebrate genomes used for conservation correspond to 7 or 10 other species: bosTau4 (*Bos Taurus*), canFam2 (*Canis familiaris*), mm9 (*Mus musculus*), monDom5 (*Monodelphis domestica*), panTro2 (*Pan troglodytes*), rheMac2 (*Macaca mulatta*), rn4 (*Rattus norvegicus*), danRer6 (*Danio rerio*), galGal3 (*Gallus gallus*) and tetNig2 (*Tetraodon nigroviridis*). The three last genomes are not included when only 7 species are considered for conservation.

#### 3) Motif scoring

TFBS are often organized in homotypic clusters in human [80]. We used Cluster-Buster as CRM prediction method based on previous benchmark results [81], [82], although other Hidden Markov Model implementations would yield similar results, as shown for SWAN in *Drosophila* [29]. The parameters used for Cluster-Buster are the default parameters, except the –c parameter is set to zero to allow a score for every region. The Cluster-Buster score is a log likelihood ratio corresponding to log [prob(sequence given that it is a cluster of real sites)/prob(cluster sequence given that it is random DNA)]. All regions are ranked according to the Cluster-Buster score, for each species separately. These rankings are combined by rank aggregation using a probabilistic method, *OrderStatistics*, to evaluate the probability (q-value) of observing a particular configuration of ranks across the different related species by chance [35]. This results in a q-value for each region based on the species specific ranks and thus effectively integrates all comparative genomics information in a single ranking for each PWM in our library, thereby allowing for motif movement within each region. The final rank of a gene is determined by the highest rank of its best region in the cross-species ranking. Genes with a score of zero are randomly queued. Note that this motif scoring strategy has been validated and used successfully in previous implementations designed for *Drosophila*, namely cisTargetX [29] and i-cisTarget [28].

### Track-based rankings of human genes

As in the case of motif detection, TF ChIP-Seq track detection also relies on an offline scoring step whereby every gene in the human genome is scored with *M* sets of ChIP-Seq peaks (broad or narrow), generating a database of *M* ranked lists of genes, each with the most likely genomic targets of a TF at the top of the ranking.

#### 1) Regulatory track collection

The collection of TF ChIP-Seq tracks is comprised of 999 tracks from ENCODE [23], 117 from Taipale laboratory [24] and 2 in-house tracks from this study (ChIP-Seq against p53 in MCF-7 after nutlin stimulation, and input). Concerning the ENCODE tracks, all the replicates were used if available.

#### 2) TF ChIP-Seq scoring

Regulatory regions around the genes (for the three delineations, see above) were scored with the entire collection of TFBS ChIP-Seq tracks. For the scoring we used the maximum score of broad and narrow peaks (signalValue column in bed file format) within the region. Finally, each gene has one score per track. All the regions are ranked according to the scores. Note that the regions having no overlap with a peak are ranked randomly at the end of the ranking.

### Calculating motif and track enrichment on a gene set

Our motif enrichment analysis differs from standard gene set enrichment methods such as GSEA, which uses Kolmogorov-Smirnov statistics [71]. In our method, we calculate the top enrichment of a single gene set over N_motif_ genomic rankings while gene set enrichment methods assess the significance of many gene sets for one genomic ranking. Enrichment is determined by the Area Under the Recovery Curve (AUC) of the cumulative recovery curve for the input set, along the whole-genome ranking. As we are mostly interested in highly ranked genes, the AUC is computed in the top of the ranking (default set to 3%, see Fig. S2B for validation) for all PWMs or tracks of the collection. A Normalized Enrichment Score (NES) for a given motif/track is computed as the AUC value of the motif/track minus the mean of all AUCs for all motifs (or tracks), and divided by the standard deviation of all AUCs. When the distribution of AUCs follows a normal distribution then the NES score is a z-score indicative of the significance. The default NES cutoff in iRegulon is 3.0, corresponding to FDR between 3% and 9% (Fig. S3).

### Detection of the target genes

For each enriched motif, the candidate targets are selected as the optimal subset of highly ranked genes compared to the genomic background and to the entire motif collection as background. This step is illustrated in Fig. 1B. The target gene recovery is plotted along the whole-genome ranking for a given motif (blue curve) and compared to the average recovery + (2× standard deviation) (red curve) for all motifs in the collection. Similarly to the GSEA approach [71], the leading edge corresponds to the rank where the difference between the signal (blue curve) and the background (red curve) is maximal within the top ranked genes (the latter is defined by the Rank Threshold parameter). The input genes that have a better ranking than the rank at the leading edge are predicted as target genes for the given motif or track.

### Detection of TFs using Motif2TF mapping

Enriched motifs are linked to candidate TFs, which could potentially bind to the motif. If we use only the direct annotations, only a small fraction of motifs (20%) can be associated to human TFs (521 TFs with “6K” collection, 944 TFs with “10K” collection). We developed a database, which we term the *motif2TF* database, corresponding to a network of associations between motifs and TFs where motif-TF edges correspond to all motif-TF direct annotations (from different species), TF-TF edges are defined by homology (using Ensembl Gene Trees [43], [44]), and motif-motif edges are defined by motif similarity, defined by the Tomtom p-value [45]. For each motif all possible TFs are associated following different paths in the motif2TF network. In the plugin at the client side, these TFs are ranked, prioritizing directly annotated TFs, then the TF present in the input set, then the ones that are found by gene homology and finally the TFs found using motif similarity. Figure 1C illustrates the different possible paths on a motif2TF subnetwork. Motif M1 is not directly annotated to any TF (so it can be part of the unknown motif collections), but is similar to two other motifs, namely M3 and M4, both of which are directly annotated. Motif M4 is directly annotated to a human TF (TF1), while M3 is a motif annotated for a TF from another species (TF7). Three TFs in human (TF1, TF8, TF6) are possible orthologs of TF7. In this example, the link between M1 and TF1 would go via the path through M4, which is the shortest and best path (rather than via M3 and TF7). For M1, motif2TF returns TF1, TF6, and TF8 as candidate TFs, which are subsequently ranked. The second example is for motif M2 which is annotated for TF5 in another species. Three human transcription factors (TF2, TF3, TF4) are possible orthologs of TF5, which may represent for example a family of homologous TFs such as GATA factors, E2F factors, or ETS factors. In such a TF family, the consensus motif may indeed be shared by multiple family members and therefore iRegulon may return multiple or all family members as candidates. When multiple TFs are returned, we give priority to a TF when it is part of the input genes. In this example, TF2 will be preferentially associated to M2 as it belongs to the input genes (encoded by TG5 in the Figure).

### ENCODE and Factorbook ChIP-Seq datasets

ChIP-Seq data was downloaded as hg19 aligned bed files (view = peaks) from the TFBS ENCODE collections available from the following servers: http://hgdownload.cse.ucsc.edu/goldenPath/hg19/encodeDCC/wgEncodeSydhTfbs/ http://hgdownload.cse.ucsc.edu/goldenPath/hg19/encodeDCC/wgEncodeHaibTfbs/ http://hgdownload.cse.ucsc.edu/goldenPath/hg19/encodeDCC/wgEncodeUchicagoTfbs/. Almost one thousand files (999) were downloaded corresponding to 160 sequence-specific TFs (TFSS): 672 files for HAIB (Hudson Alpha Institute), 323 for SYDH (Stanford/Yale/USC/Harvard) and 6 files for Uchicago. Files corresponding to Input and RNA Polymerase 2 (“Pol2”/“Pol2(phosphoS2)”) were not downloaded. 115 TFs are detectable in iRegulon (i.e., at least one motif in the collection of 6383 motifs can be connected to the TF), corresponding to 786 ENCODE datasets. Each query set consists of the top 200 target genes presenting a ChIP peak in a predefined search space, i.e., for each search space tested (500 bp upstream of TSS; 10 kb around TSS; 20 kb around TSS), we define a different set of target genes, so that each target gene contains a ChIP peak within the chosen motif search space. The ChIP-Seq scoring of the genes has been done as mentioned earlier in the *Track-based rankings* section. Finally, note that our motif collection does not contain PWMs derived from these datasets (so we rely on other, previously curated PWMs to identify the correct TF). The Factorbook dataset collection is a subset of this ENCODE selection corresponding to 254 ChIP-Seq files (121 from HAIB, 129 from SYDH and 5 from Uchicago), inferred from the list of signatures published in the Table S1 of the FactorBook reference publication [46]. 126 out of these 254 FactorBook signatures have the canonical motif corresponding to the ChIP'ped TF. From these we randomly selected one signature per TF for which the canonical motif was predicted as top 1 by their motif discovery pipeline (inferred from Table S1A [46]). The list of the 30 used datasets is presented in Table S3. Different types of control gene sets were selected, namely: from ENCODE ChIP-Seq we used (1) genes without a ChIP-Seq peak in the corresponding search space; (2) TF neighborhoods for 1150 TFs, containing for each TF all the genes within 5 Mb flanking the TF; and (3) 1161 random signatures. Datasets are available on our laboratory website (http://www.aertslab.org). We also got similar performances using 631 uniformly reprocessed ChIP-Seq data generated in NarrowPeak format by the ENCODE Analysis Working Group downloaded from http://genome.ucsc.edu/cgi-bin/hgFileUi?db=hg19&g=wgEncodeAwgTfbsUniform (data not shown).

### Selection of other tools used for comparison with iRegulon

The classical motif discovery algorithms that originated in the late 1990s can be put in two categories: string-based or enumeration methods and matrix-based approaches. The string-based approaches rely on the detection of statistically over-represented words (oligonucleotides or spaced motifs) compared to a given background [83]–[88]. Matrix-based approaches make use of position weight matrices (PWMs) as a predictive model for TF binding sites, which can be graphically represented as a motif logo [89], and use optimization algorithms (Expectation-Maximization [90], greedy algorithm [91], [92] or Gibbs sampling [93]–[95]) to find the most common motifs to all input sequences. Most of these methods performed well on yeast or bacterial promoter sequences, but they showed limited performances when applied to mouse or human [96]. These methods could be improved by phylogenetic footprinting [97]–[103] and by applying genome-scale methods that exploit the entire gene expression data set rather than a set of co-expressed genes [104]–[106]. Current developments have on the one hand focused on the application of the early algorithms to ChIP-Seq data [107]–[111], and on the other hand on the application of motif discovery to gene sets, with the aim to increase the performance in higher eukaryotes such as fly, mouse and human, using large sequence search spaces. This category of PWM enrichment methods is represented by phylCRM/Lever [30], DIRE [80], [112], PASTAA [32], [113], PSCAN [114], Allegro [115], HOMER [116], OPOSSUM [117] and i-cisTarget [28]. They all use libraries of candidate PWMs and apply PWM enrichment statistics, often combined with other cues, such as comparative genomics and TF binding site clustering. By using libraries of PWMs for known TFs (e.g., PWMs derived from protein binding microarrays), these methods promote a TF to a candidate master regulator of the gene set when its PWM is found enriched. We used all methods in this category of PWM enrichment methods that are available online, that can work on human gene sets, and that can be practically performed on 30 sets of 200 human genes.

### Benchmark analysis

Thirty gene sets from FactorBook were selected for motif discovery tool comparison (Fig. 2D, Table S1). These gene sets have been selected because the motif of the ChIP'ped TF was detected as top enriched motif in the top 500 peaks in FactorBook. We extracted the top 200 genes having the highest peaks in their 20 kb region around the TSS. The comparison was performed on TF and motif recovery using the parameters indicated in Table S3. The parameters were left to default and when possible, we only adjusted the parameters to allow for larger upstream regions (when possible we choose TSS+−10 kb). iRegulon was compared to eight other publicly available motif enrichment tools, namely OPOSSUM [117], DIRE [80], [112], PASTAA [32], [113], PSCAN [114], Clover [16], AME [118], Allegro [115] and HOMER2 [116] (in the case of Homer2, *de novo* and known motif discovery are performed simultaneously but we consider them as different approaches and validate them separately). We selected these tools because they mostly take as input a set of human co-expressed genes, and they all return, at least to some extent, information on which TF could be regulating the input genes. For this reason, it not feasible to compare iRegulon with classical *de novo* motif discovery methods (e.g., MEME-like methods) because such methods are intractable on large human gene sets (e.g., 200 genes×20 kb×10 species represents a sequence set of 40 Mb), and they result in new motifs rather than candidate TFs. We also attempted to use SMART [119] but we did not succeed in running the software. For tools that require regulatory sequences as input (AME and Clover) we used the same sequences as used by iRegulon. For some tools like Clover, it is theoretically possible to use a large search space but one run on one dataset takes too long (∼17 hours), and therefore we limited the analysis to 500 bp promoter sequences. In the case of AME, we found no positive results with a large search space (data not shown), so we show the results with the default search space. For comparison, we used the number of motifs/TFs found in top 1 and within top 5 positions. The total number of detected motifs was not reported for comparison, because some tools use more stringent thresholds than others. All these tools rely on the available motif annotation to identify the candidate TF such as Jaspar (J) or Transfac (T). However, we also manually re-associated the detected motifs to candidate TFs (mainly by comparison of the detected motif with the FactorBook motif) (see column “USING SIMILARITY” in the Table S3). For Homer2, 14 motifs that are derived from ENCODE ChIP-Seq data matching the actual Factorbook ChIP-Seq data were discarded from their in-house PWM collection to avoid over-fitting (indeed, iRegulon does not include FactorBook PWMs either, nor do any of the other tools). Note that for the other large-scale analysis (e.g. full ENCODE analysis), we use a command-line version of iRegulon.

### iRegulon Cytoscape plugin and server-side daemon

At the client side, iRegulon is implemented in JAVA as a *Cytoscape* plugin, which can be downloaded from http://iregulon.aertslab.org. The iRegulon plugin is connected to the server-side *daemon* over the Internet. The iRegulon server-side daemon is implemented in Python and uses MySQL to store and query the PWM-based whole-genome rankings (see below). After submitting a gene set or network to the service, the results are returned to the client, and this happens on-the-fly, and takes about one minute. The user can browse through the motif discovery results, select a TF among the prioritized list of TFs, and add upstream regulators and direct regulator-target ‘edges’ to the input gene set or network under study. A detailed description on the use of the plugin is provided in Figure S4. In addition, the plugin allows querying *cisTargetDB* to obtain the meta-regulon for a given TF, i.e. targets found recurrently predicted for this TF by iRegulon across thousands of signatures/gene sets. iRegulon results were obtained by running the Cytoscape plugin v0.97 on Cytoscape 2.8.1. The current version of iRegulon (1.2) supports the “10K” motif collection and the track discovery. The source code of the iRegulon plugin is also available from the iRegulon website (http://iregulon.aertslab.org).

### A database with meta-regulons

iRegulon was applied in batch (i.e., using the GMT file format as input for the command line version of iRegulon) to 3447 signatures in GeneSigDB (version 4), 6753 signatures from MSigDB (version v3 collection 2) and 12972 bi-clusters we obtained in-house. Bi-clustering was performed with Ganesh clustering algorithm [120], [121] using default settings to 91 microarray datasets. The 91 datasets were retrieved as normalized (fRMA) microarray data from InsilicoDB [122]. iRegulon results on all these gene sets is stored in a MySQL database, from which all summaries per motif and subsequently per TF are computed, resulting in a meta-regulon per TF. In a meta-regulon, each target gene is annotated with a number that represents the number of gene sets where the TF is found enriched *and* the gene is among the optimal subset of direct targets.

### Gene Ontology (GO) and GSEA enrichment analysis

GO enrichment analysis was performed using DAVID [123], [124] or BINGO [125]. GSEA analysis on ChIP-Seq data was performed to avoid arbitrary peak score cutoffs. The genome was ranked according to the MACS ChIP-peak score (score range between 0 and 1517.33 for p53) within an area of 20 kb around the TSS of 22284 RefSeq genes. Functional categories found enriched for co-factors of p53 were calculated by DAVID and WebGestalt [126] based on Gene Ontology and KEGG pathways.

### Culturing of MCF-7 cells

Cells were kept in culture at 37°C, with 5% CO2 and in RPMI medium (+ L-glutamate, Gibco) supplemented with 10% fetal bovine serum (Invitrogen), 0.4 mM sodium pyruvate (Gibco), 100 µm/ml penicillin/streptomycin (Invitrogen), 1× non-essential aminoacids (Gibco) and 10 µg/ml Insulin (Sigma).

### RNA-seq

p53-Wild-Type MCF-7 cells were plated onto 24-well plates (60000 cells/well). The next day, cells were either stimulated with 5 µM Nutlin-3a or left untreated. After 24 h, cells were washed in PBS (Gibco) and prepared for RNA extraction according to the RNeasy protocol (Qiagen), yielding around 2 µg of total RNA per sample. The quality of the RNA samples were checked using a Bioanalyzer 1000 DNA chip (Agilent) after which libraries were constructed according to the Illumina TruSeqTM RNA Sample preparation guide. Final libraries were pooled and sequenced on the HISeq 2000 (Illumina), generating approximately 30 million reads of 50 bp length. After removing adapter sequences reads were mapped to the human reference genome (hg19) using TopHat v1.3.3 [127] with default settings. Reads were aggregated with HT-Seq (–str = no parameter, version 0.5.3p3) using the human RefSeq annotation, release 42. DESeq [128] was used to normalize and to calculate differential expression between Nutlin-3a stimulated and non-stimulated samples. A final list of differentially expressed genes was obtained using adjusted p-value<0.05 and |log2FC|>1. The threshold of 2-fold up-regulation was supported by the observation that the strongest enrichment of the targets from the KEGG p53 signaling pathway is observed among the top 648 up-regulated genes (GSEA leading edge corresponds to log2FC = 1.182).

### ChIP-Seq

p53 wild-type MCF-7 cells were seeded at a density of 5 million cells per 15 cm dish and grown ON at 37°C to 80–90% confluency. Cells were then stimulated with 5 µM Nutlin-3a for 24 h. ChIP samples were prepared following the Magna ChIP-SeqTM preparation kit using the p53 antibody (DO-1, SCBT). Per sample, 5–10 ng of precipitated DNA was used to perform library preparation according to the Illumina TruSeqTM DNA Sample preparation guide. In brief, the immunoprecipitated DNA was end-repaired, A-tailed, and ligated to diluted sequencing adapters (dilution of 1/100). After PCR amplification with 15–18 cycles and gel size selection of 200–300 bp fragments, the libraries were sequenced using the HiSeq 2000 (Illumina). Cleaned reads were mapped to the human reference genome hg19 (UCSC) using bowtie (v2.0.0-beta3) with the addition of parameter –local, allowing for further soft clipping of the reads. Reads with a mapping quality below 4 were removed. Peak calling was performed using MACS (version 1.4.2) [129] either with the default p-value threshold (3634 peaks) of 1.0E-5 or using p-value<0.05 (lenient setting to generate the whole-genome ranking).

### RT-qPCR

MCF-7, HCT116 (human colon carcinoma cell line) and BJ cells were treated continuously with 10 µM Nutlin-3a or a pulse of 5 µM Doxorubicin and total RNA was harvested at different time points. Reverse transcription was performed using the High Capacity cDNA Reverse Transcription Kit (Applied Biosystems). Real Time quantitative PCR reactions were run on LightCycler480 (Roche) in 384-well format, using SYBR-Green Fast Universal PCR Master Mix (Applied Biosystems). Multiple primer pairs were tested for each target, and melting curve analysis confirmed amplification of a single product. Normalization was done with the most stable reference genes, assessed by GeNorm analysis [130]. The normalized relative fold changes were log-transformed before performing two-sided t-test to determine significance levels. The p-values were further corrected for multiple testing by very stringent Bonferroni correction. RT-qPCR primer sequences: NHLH2-fw-CACTGTGGGAGGATCTGAGC; NHLH2-rev-ATAAAGGGGCACTTCGCCTG; ALDH3A1-fw-CTGCAGGGAACTCAGTGGTC; ALDH3A1-rev-GGTACAGATCCTTGTCCAGGT; SLC12A4-fw-GGGAACAACATTCGCAGCAG; SLC12A4-rev-AGTGGCATTCGACGTGTCAT; RAP2B-fw-GCGCACAAAAGCCAAACGC; RAP2B-rev-AGACACCCTGGCCAATGCAA.

### Transfection and luciferase assays

MCF-7 cells (WT or p53-KD) were seeded in a 24 well plate at a density of 50 000 cells per well. After 24 h, cells were transfected using Fugene HD (Promega) in a 1∶3 ratio. 400 ng of luciferase reporter plasmid containing one of the enhancers of interest (CDKN1A, RAP2B, ALDH3A1, SLC12A4 and NHLH2) was mixed with a β-gal plasmid in a 1∶10 ratio to correct for transfection efficiency. The next day, cells were stimulated with 5 µM Nutlin-3a. After 24 h, the transfected cells were harvested and luciferase and β-galactosidase activities were measured following the manufacturer's instructions (Applied Biosystems). The p-values were calculated using a t-test.

### Accession numbers

The RNA-Seq and ChIP-Seq data are available from the NCBI GEO database (GSE47043).

## Supporting Information

Figure S1 **Delineation tracks for CDKN1A transcripts in the human genome.** UCSC Genome Browser Gateway screenshot showing the human genome (hg19) region around the CDKN1A loci (chr6:36644237–36655116). The top tracks show our different delineations: in green (500 bp upstream of the TSS, named “500 bp”), in pink (TSS+−5 kb, named “5 kb”) and in blue (TSS+−10 kb, named “10 kb”). The screenshot also shows different tracks (from top to bottom): the Refseq genes annotations, the mark of active chromatin (H3K27Ac) from ENCODE, the density of DNaseI Clusters from ENCODE, the density of Transcription Factor ChIP-Seq from ENCODE, the CpG islands, the regulatory elements annotated in OregAnno, and vertebrate basewise conservation by PhyloP. The promoter (and sequences further upstream and downstream of TSS) of each alternative transcript is used. This can be seen when we consider the delineation of the 500 bp promoters, depicted as green track in the figure. In the RefSeq annotation there are two major TSSs, and each has its own promoter. However, in the large search spaces the respective upstream and downstream regions of both TSSs overlap, and become one large merged region (pink and blue for 5 kb and 10 kb respectively).
(TIF)

Figure S2 **iRegulon validation and tool comparison.** **A.** Motif2TF validation. Recovery for ENCODE signatures and their control sets using different motif2TF parameters: 1) Motif collection effect (J, T, A barcharts), 2) Homology effect using threshold on Identity% for all motifs (A+O barcharts), 3) Motif similarity effect using threshold on the p-value (A+S barcharts), and combinations (A+O+S). Only Jaspar motifs (J); Only Transfac Pro (T); All motifs from Jaspar and Transfac pro, and others databases (A); All motifs+Orthology (A+O); and All motifs+Orthology+Similarity (A+O+S); blue indicates the analysis done on ENCODE sets and grey indicates on the control sets. The color (from red to yellow) and order of stacked bars indicate the number of times the queried TF was identified in the 1^st^ rank (top1), 2^nd^ rank (top2), 3^rd^ rank (top3), 4^th^ rank (top4), 5^th^ rank (top5) and 6^th^ to 10^th^ rank (top10). White color indicates the number of detected TFs (motif enrichment ≥3) but with rank >10. By comparing several combinations of different thresholds on orthology and motif similarity, we propose to not use any threshold on the percentage of identity (i.e., using any homologous relationship); and to use a stringent threshold (p-value<0.001) on the motif similarity to avoid the high false discovery rates in random control sets (labeled as “A+O[0%]+S[0.001]” in the plot or “A+O+S” in Figure 2C). **B.** Validation of the rank thresholds for the AUC calculation. The performances are quite robust to variation to the rank thresholds within a range of 0.01% to 0.3%, but note that the larger this threshold the longer the computation time. **C.** TF recovery for Factorbook (similar as Fig. 2D) but results of tools using JASPAR motif collection only. Tool comparison using top 200 genes showing a top peak in their 20 kb regulatory region from 30 ENCODE ChIP-Seq having a top motif identified for the ChIP'ped TF in Factorbook (see Table S1). Default parameters were used, but when possible, they were adjusted to use the tss-centered-20 kb regions. iRegulon was run without the use of motif2TF and restricted to Jaspar motif collection only (“motif”) or with the use of motif2tf (“motif2tf”). **D.** Target recovery using two different search spaces: a proximal region (TSS-up500 bp) *versus* a large region (TSS+−10 kb) for each gene set in the 30TF collection selected from Factorbook. The proportion of genes with proximal peaks have been calculated for each TF by the overlap between the inferred gene sets with peaks found within the proximal search space (all genes) and the large search space (top 200 genes). Overall, when iRegulon is applied on a 20 kb search space (TSS+−10 kb), more true target genes are identified (i.e., higher sensitivity shown as green bars), compared to iRegulon on 500 bp promoter only.
(TIF)

Figure S3 **FDR plots for each regulatory search space.** The plots in A, C, E shows the TF recovery (y-axis) on the ENCODE ChIP-Seq datasets (in blue) for a given NES threshold (x-axis) and a given regulatory search space, and the TF recovery found for the same delineation on the control ENCODE sets (bottom ranked genes) (in green). The plots in B, D, and F panels show the FDR calculated by comparing the ratio of the TF recovery in control datasets over the TF recovery in biological datasets (ENCODE ChIP-Seq). For NES> = 3, the FDR is between 1% and 5% for the delineation of 500 bp upstream the TSS (up500 bp) (A,B), between 8% an 9% for TSS+−10 kb (C,D), and between 6% and 7% for TSS+−20 kb (E,F).
(TIF)

Figure S4 **Description of the iRegulon Cytoscape plugin.** Panels A–E show the prediction of master regulators and targets and panels F–G show the query of meta regulons predicted from the systematic iRegulon analysis on thousands of cancer gene signatures. **A.** Input network. To perform TF and target predictions, the initial gene set can be a set of selected nodes in an existing gene network in Cytoscape or can be imported from a text file using the menu *File > import network as a table*. **B.** The query form presented here allows the user to give a name to the analysis, specify the gene nomenclature, and choose the motif and the track collection, the type of search space (gene-based or region-based), the regulatory search space (500 bp upstream of the TSS, 10 kb or 20 kb around the TSS) and the conservation (within 7 or 10 species). The motif prediction parameters are the enrichment score threshold, the ROC threshold for AUC calculation, and the Rank threshold for target selection. The TF prediction parameters are the minimal percentage of identity and the maximal FDR for motif similarity. Then, it is possible to choose for the node attribute having the gene IDs (HGNC symbols), and the number of selected nodes is displayed. **C.** Results panel (motif view). The raw results correspond to a list of enriched motifs, together with a prioritized list of candidate transcription factors that can bind the motif. The main table shows the motifs ranked by decreasing NES score, with the calculated AUC, the number of predicted targets (#Targets) and the number of TFs (#TF) found by *motif2TF* mapping. Note that when the number of TFs is zero it means that the motif cannot be associated to a known TF, but can still be detected as enriched. The enriched motifs are clustered by STAMP [137] so that similar motifs are visually represented with different colors in the Results table. The sub-table is related to the selected motif (highlighted in blue background) and shows: 1) on the left side, the associated TF(s) with the value of the evidence parameters (Motif similarity and %identity); 2) on the right side, the corresponding predicted targets with their rank for this motif. **D.** Results panel (track view). The top table shows the enriched tracks ranked by the maximal NES score, presented with the number of merged targets (#Targets). The sub-table shows the track description on the left side. The mid-table shows the ChIP'ped TF. The table on the right side shows the ranked targets. **E.** Results panel (transcription factor view). The top table shows the enriched TFs ranked by the maximal NES score, presented with the number of merged targets (#Targets) found by numerous motifs/tracks (#Motifs/Tracks). The sub-table shows the motifs or tracks results for a selected TF on the left side. The mid-table shows the predicted TFs that can be associated by motif2TF to these motifs with the levels of evidence (%identity, motif similarity and number of motifs). The table on the right side shows the ranked targets and the number of motifs for which they are predicted. In this example, iRegulon has been applied to 171 genes that are up-regulated in MCF-7 cells under hypoxia conditions. These genes come from the curated signature named “ELVIDGE_HYPOXIA_UP” in MSigDB (C2 CGP). The highest-scoring regulon contains HIF1A as master regulator. **F.** The output network for HIF1A can be drawn by clicking on the button “+” (“Add regulator and target genes with their interactions to the current network”). iRegulon parameters are 20 kb around the TSS (7 species), ROC threshold: 0.03, minimum orthologous identity: 0%, FDR for maximum motif similarity: 0.001. **G.** Query panel of TF-target database. To query the database of meta-regulons, the user needs to go to the query form using Cytoscape menu (*Plugins > iRegulon > Query TF-target database*). The query form allows the user to select the TF and the Species, and the databases of signatures/gene sets (GeneSigDB, Ganesh clusters or/and MSigDB). The occurrence count threshold indicates the minimal number of signatures, and the second parameter indicates the maximal number of nodes to display in the network. Then, it is possible to choose for the node attribute having the gene IDs (only HGNC symbols are supported), and to tick the box to automatically create a new network. **H.** Output network resulting from the query of TF-target database (F).
(TIF)

Figure S5 **Regulons are detected in many types of networks and gene sets.** iRegulon can be applied to any kind of gene set to predict upstream regulatory TFs along with significant direct targets, forming TF-target *regulons*. **A.** 94 HIF1alpha targets identified in 171 genes involved in Hypoxia (11 PWMs, NES = 4.89, rank = 1) (see also Fig. S2 for further details on this iRegulon analysis). Known HIF1A targets [54] are in thick circles. **B.** Application to genes from the Notch signaling pathway (Pathway Commons Web Service Client in Cytoscape: NCI/Nature Pathway Interaction Database (ID: notch_pathway)). The imported pathway is composed of 161 molecules and 750 edges. Pathway interactions between genes are in grey and predicted regulatory interactions are in green or blue. We applied iRegulon on all the 87 genes. *HES1* (green edges source node) is ranked 1st (NES = 5.099, 5 PWMs) with 35 predicted direct targets. *RBPJ* (blue edges source node) is ranked 3rd (NES = 4.329, 2 PWMs) with 17 predicted direct targets, including *HEY1*, *HEY2*, and *HES1*. These co-regulators control 47% of the genes if the NOTCH signalling pathway (41/87 genes). **C.** Application to immune response signature. The Immune response gene set is a list of 1923 gene products in Homo sapiens associated to immune response (GO:0006955 and children) was downloaded as a tab delimited file from http://amigo.geneontology.org. Then, this list was converted in a list of 1198 unique gene names (HGNC) and imported in Cytoscape as a network. When applied to these 1198 genes, iRegulon finds the IRF and REL/NFkB regulons, with 806 and 711 direct target genes respectively, indicating that these are indeed that master regulators of the immune response. **D.** Application to protein-protein interactions from STRING. iRegulon was applied to 500 genes associated with p53 in STRING. The p53 motif was found enriched with an enrichment score of 4.59. Predicted direct interactions are shown in red. **E.** Application to microRNA targets. iRegulon analysis has been performed on 159 microRNAs with annotated targetomes. Examples are shown for annotated targets of hsa-miR-133a, has-miR-32, has-miR-429 and has-miR-106a. microRNAs are in red nodes and target nodes are in blue or red (TF). For each microRNA targetome, the enriched TF (found by iRegulon) is represented in green. For example, SRF (green node) was found enriched with a top motif ranked 5^th^ (NES = 4.149) in hsa-miR-133a targetome.
(TIF)

Figure S6 **Validation of predicted regulons.** **A–C.** PeakMotifs results. (A) Results of peakMotifs when applied on peaks near genes that are NOT predicted as direct p53 targets by iRegulon. On this set the p53 motif is not found. (B) Results on the ChIP peaks of up-regulated genes that are also direct targets. On this set of peaks the p53 motif is clearly found. (C) Results on the peaks near down-regulated genes, again not finding the p53 motif. **D.** GSEA results validating the iRegulon E2F predicted targets with E2F1ChIP-Seq results. Both the total set of down-regulated genes and the predicted E2F direct targets are highly enriched. E2F ChIP-Seq data in the same MCF-7 cell line were downloaded as fastq files from ENCODE. The sequences were mapped to hg19 using same mapping parameters as for p53 ChIP-Seq experiments and the bam files of the replicates were merged with samtools. See Experimental Procedures for the description of the peak calling and ranking of the genes. ENCODE Ids: wgEncodeYaleChIPseqRawDataRep1Mcf7Hae2f1, wgEncodeYaleChIPseqRawDataRep2Mcf7Hae2f1, wgEncodeYaleChIPseqRawDataRep1Mcf7Input, wgEncodeYaleChIPseqRawDataRep2Mcf7Input.
(TIF)

Figure S7 **Gene Set Enrichment Analysis (GSEA) on GeneSigDB Meta-regulons.** **A**. p53 meta-regulon (188 genes, min 3 signatures) is found positively enriched by GSEA on the preranked list of genes weighted by our in house p53 ChIP-Seq peak scores with a NES of 3.01, Nominal p-value = 0, FDR q-value = 0, leading edge at 890th rank of the signature. (B–E) GeneSigDB meta-regulon for TFs found enriched in ENCODE ChIP-Seq data using GSEA with 516/827 gene sets that passed the gene set size filters (min = 15, max = 1000) and corresponding to 78 TFs used in ENCODE ChIP-Seq datasets. **B**. ZEB1 meta-regulon (46 genes) is found positively enriched with a NES of 1.24, Nominal p-value = 0.001, FDR q-value = 0.918, leading edge at 950th rank of the signature. **C**. CREB1 meta-regulon (512 genes) is found positively enriched with a NES of 1.07, Nominal p-value = 0, FDR q-value = 1, leading edge at 3069th rank of the signature. **D**. FOXA2 meta-regulon (410 genes) is found positively enriched with a NES of 1.21, Nominal p-value = 0, FDR q-value = 0.191, leading edge at 3069th rank of the signature. **E**. CTCF meta-regulon (57 genes) is found positively enriched with a NES of 1.48, Nominal p-value = 0, FDR q-value = 0.11, leading edge at 6353th rank of the signature. Signature IDs are wgEncodeHaibTfbsGm12878Zeb1sc25388V0416102PkRep2 (B), wgEncodeHaibTfbsEcc1Creb1sc240V0422111PkRep2 (C), wgEncodeHaibTfbsA549Foxa2V0416102Etoh02PkRep1 (D), and wgEncodeSydhTfbsK562CtcfbIggrabPk (E).
(TIF)

Figure S8 **Time-course experiments by RT-qPCR.** mRNA levels in log2FC of p53 target genes in MCF-7 cells after stimulation with 10 mM Nutlin3a (**A**) or 1 hour pulse of 5 mM Doxorubicin (**B**).
(TIF)

Table S1 **FactorBook gene sets used for tool comparison.**
(XLSX)

Table S2 **Up- and down-regulated genes between Nutlin stimulated (S) vs non stimulated (NS) in MCF-7, with log fold changes and adjusted p-values.**
(XLSX)

Table S3 **iRegulon results on (A) up-regulated and (B) down-regulated genes.**
(XLSX)

Table S4 **Predicted p53 targets by iRegulon and p53 ChIP peaks annotated for all the 801 up-regulated genes after Nutlin stimulation.**
(XLSX)

Table S5 **Curated p53 targets.**
(XLSX)

Table S6 **Overlap between 110 predicted p53 targets, p53 meta-regulon, and p53 targets published in recent literature.**
(XLSX)

Table S7 **iRegulon results on up-regulated and down-regulated genes using motif (10K collection) and track discovery.** Orange rows indicate enriched motifs while green rows indicate enriched tracks.
(XLSX)
