# Supplementary figures and images for: iRegulon: From a Gene List to a Gene Regulatory Network Using Large Motif and Track Collections
Source: PLoS Comput Biol. 2014 Jul 24;10(7):e1003731. doi: 10.1371/journal.pcbi.1003731 (PMC4109854; doi:10.1371/journal.pcbi.1003731)

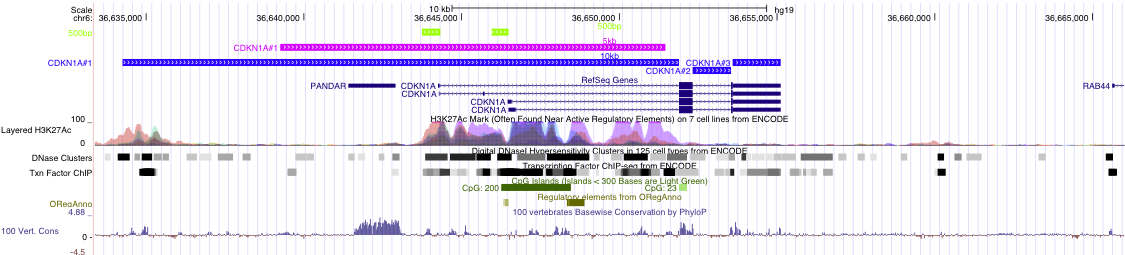

Supplement: Figure S1 — Delineation tracks for CDKN1A transcripts in the human genome. UCSC Genome Browser Gateway screenshot showing the human genome (hg19) region around the CDKN1A loci (chr6:36644237–36655116). The top tracks show our different delineations: in green (500 bp upstream of the TSS, named “500 bp”), in pink (TSS+−5 kb, named “5 kb”) and in blue (TSS+−10 kb, named “10 kb”). The screenshot also shows different tracks (from top to bottom): the Refseq genes annotations, the mark of active chromatin (H3K27Ac) from ENCODE, the density of DNaseI Clusters from ENCODE, the density of Transcription Factor ChIP-Seq from ENCODE, the CpG islands, the regulatory elements annotated in OregAnno, and vertebrate basewise conservation by PhyloP. The promoter (and sequences further upstream and downstream of TSS) of each alternative transcript is used. This can be seen when we consider the delineation of the 500 bp promoters, depicted as green track in the figure. In the RefSeq annotation there are two major TSSs, and each has its own promoter. However, in the large search spaces the respective upstream and downstream regions of both TSSs overlap, and become one large merged region (pink and blue for 5 kb and 10 kb respectively). (TIF) [file pcbi.1003731.s001.tif]

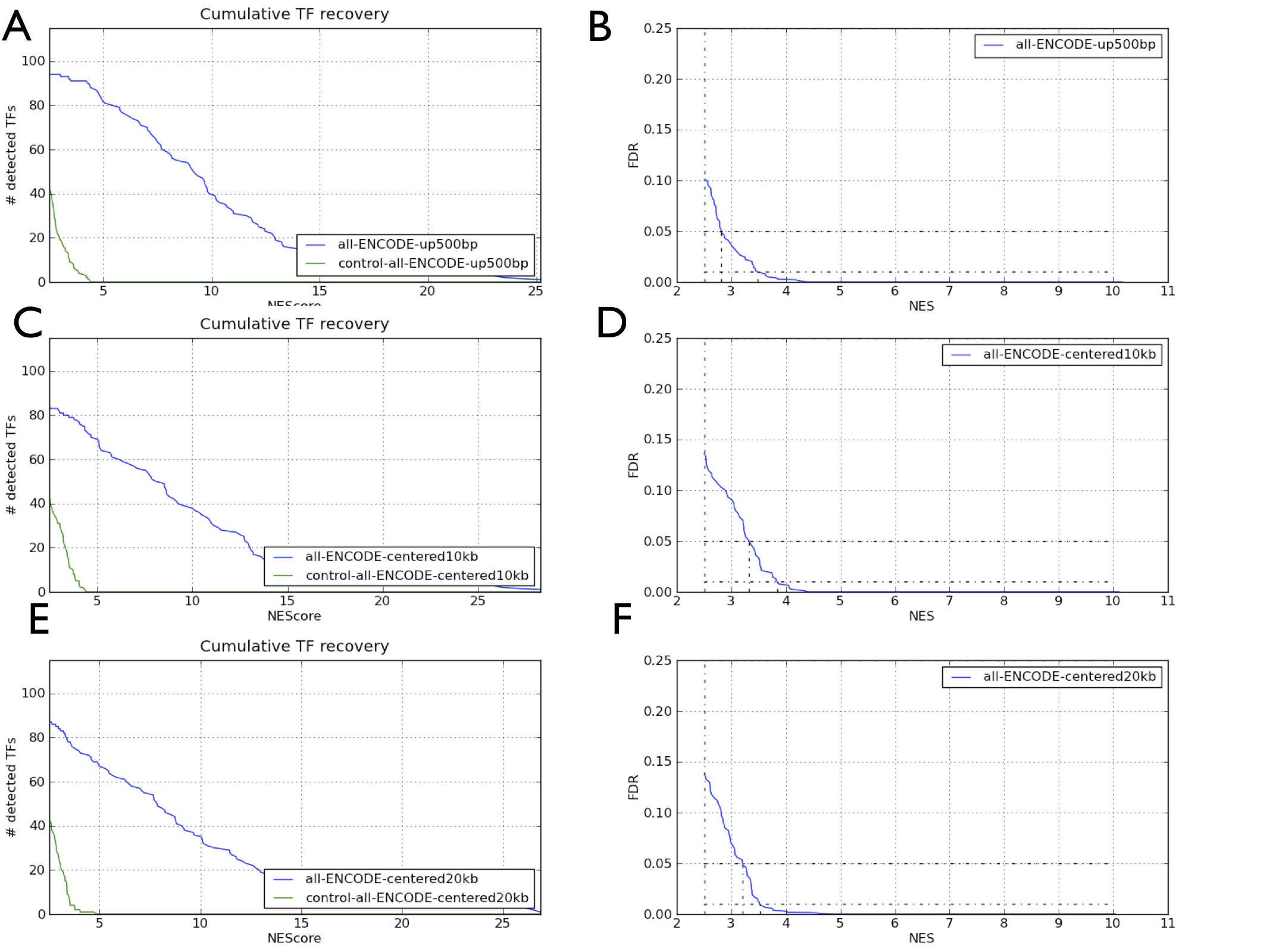

Supplement: Figure S3 — FDR plots for each regulatory search space. The plots in A, C, E shows the TF recovery (y-axis) on the ENCODE ChIP-Seq datasets (in blue) for a given NES threshold (x-axis) and a given regulatory search space, and the TF recovery found for the same delineation on the control ENCODE sets (bottom ranked genes) (in green). The plots in B, D, and F panels show the FDR calculated by comparing the ratio of the TF recovery in control datasets over the TF recovery in biological datasets (ENCODE ChIP-Seq). For NES> = 3, the FDR is between 1% and 5% for the delineation of 500 bp upstream the TSS (up500 bp) (A,B), between 8% an 9% for TSS+−10 kb (C,D), and between 6% and 7% for TSS+−20 kb (E,F). (TIF) [file pcbi.1003731.s003.tif]

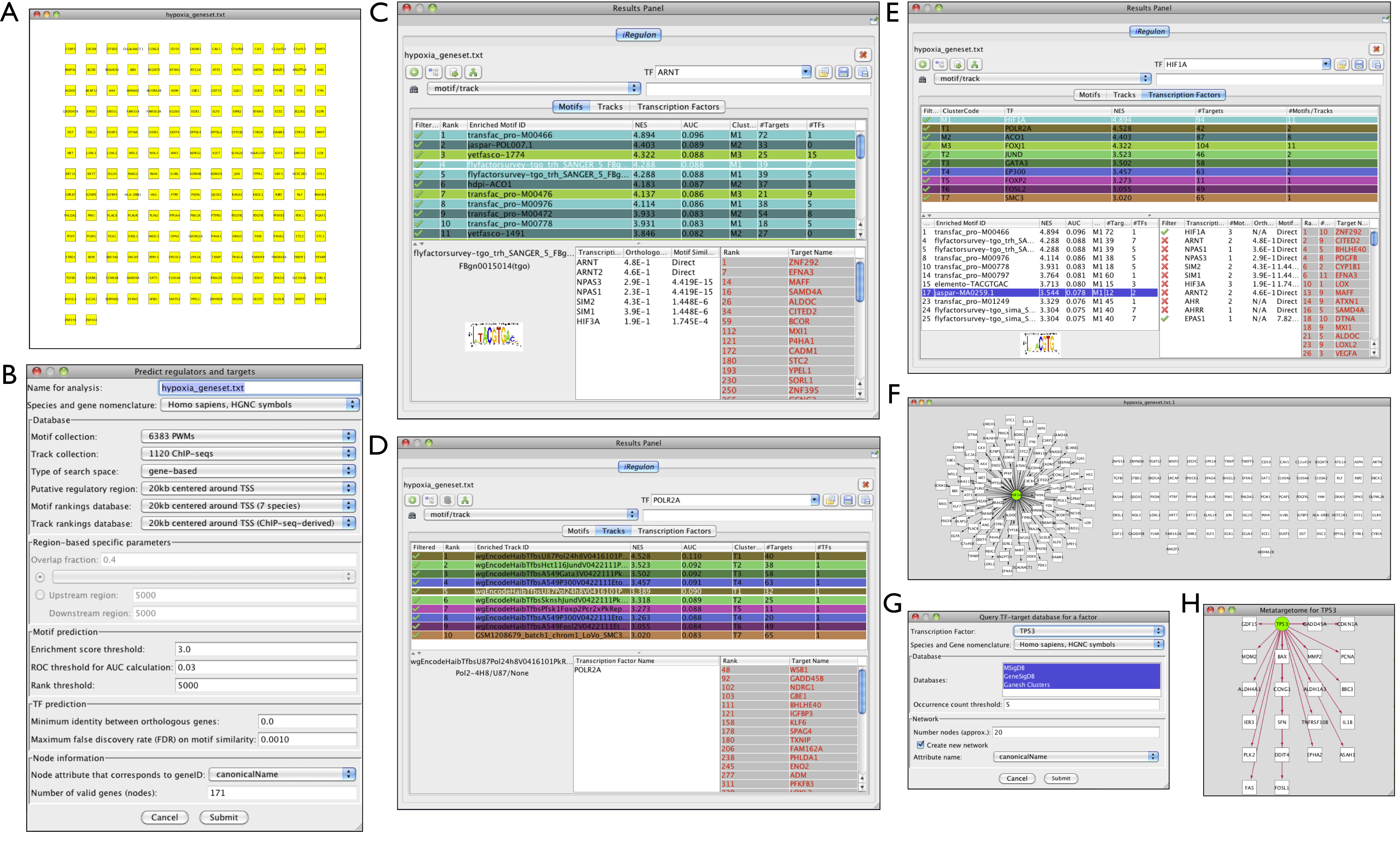

Supplement: Figure S4 — Description of the iRegulon Cytoscape plugin. Panels A–E show the prediction of master regulators and targets and panels F–G show the query of meta regulons predicted from the systematic iRegulon analysis on thousands of cancer gene signatures. A. Input network. To perform TF and target predictions, the initial gene set can be a set of selected nodes in an existing gene network in Cytoscape or can be imported from a text file using the menu File > import network as a table. B. The query form presented here allows the user to give a name to the analysis, specify the gene nomenclature, and choose the motif and the track collection, the type of search space (gene-based or region-based), the regulatory search space (500 bp upstream of the TSS, 10 kb or 20 kb around the TSS) and the conservation (within 7 or 10 species). The motif prediction parameters are the enrichment score threshold, the ROC threshold for AUC calculation, and the Rank threshold for target selection. The TF prediction parameters are the minimal percentage of identity and the maximal FDR for motif similarity. Then, it is possible to choose for the node attribute having the gene IDs (HGNC symbols), and the number of selected nodes is displayed. C. Results panel (motif view). The raw results correspond to a list of enriched motifs, together with a prioritized list of candidate transcription factors that can bind the motif. The main table shows the motifs ranked by decreasing NES score, with the calculated AUC, the number of predicted targets (#Targets) and the number of TFs (#TF) found by motif2TF mapping. Note that when the number of TFs is zero it means that the motif cannot be associated to a known TF, but can still be detected as enriched. The enriched motifs are clustered by STAMP [137] so that similar motifs are visually represented with different colors in the Results table. The sub-table is related to the selected motif (highlighted in blue background) and shows: 1) on the left side, the associat [file pcbi.1003731.s004.tif]

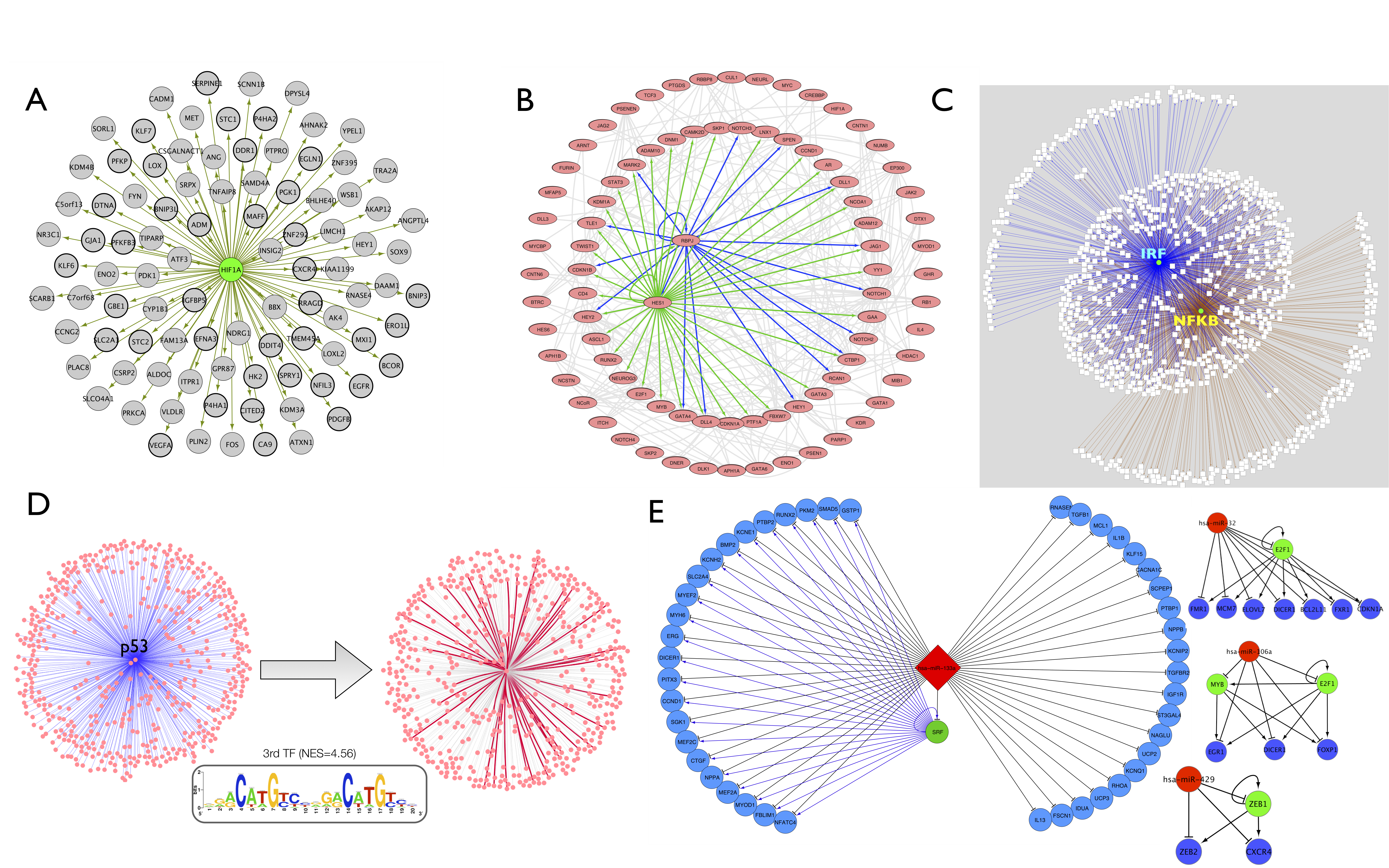

Supplement: Figure S5 — Regulons are detected in many types of networks and gene sets. iRegulon can be applied to any kind of gene set to predict upstream regulatory TFs along with significant direct targets, forming TF-target regulons. A. 94 HIF1alpha targets identified in 171 genes involved in Hypoxia (11 PWMs, NES = 4.89, rank = 1) (see also Fig. S2 for further details on this iRegulon analysis). Known HIF1A targets [54] are in thick circles. B. Application to genes from the Notch signaling pathway (Pathway Commons Web Service Client in Cytoscape: NCI/Nature Pathway Interaction Database (ID: notch_pathway)). The imported pathway is composed of 161 molecules and 750 edges. Pathway interactions between genes are in grey and predicted regulatory interactions are in green or blue. We applied iRegulon on all the 87 genes. HES1 (green edges source node) is ranked 1st (NES = 5.099, 5 PWMs) with 35 predicted direct targets. RBPJ (blue edges source node) is ranked 3rd (NES = 4.329, 2 PWMs) with 17 predicted direct targets, including HEY1, HEY2, and HES1. These co-regulators control 47% of the genes if the NOTCH signalling pathway (41/87 genes). C. Application to immune response signature. The Immune response gene set is a list of 1923 gene products in Homo sapiens associated to immune response (GO:0006955 and children) was downloaded as a tab delimited file from http://amigo.geneontology.org. Then, this list was converted in a list of 1198 unique gene names (HGNC) and imported in Cytoscape as a network. When applied to these 1198 genes, iRegulon finds the IRF and REL/NFkB regulons, with 806 and 711 direct target genes respectively, indicating that these are indeed that master regulators of the immune response. D. Application to protein-protein interactions from STRING. iRegulon was applied to 500 genes associated with p53 in STRING. The p53 motif was found enriched with an enrichment score of 4.59. Predicted direct interactions are shown in red. E. Application to microRNA targets. iRegulon analy [file pcbi.1003731.s005.tif]

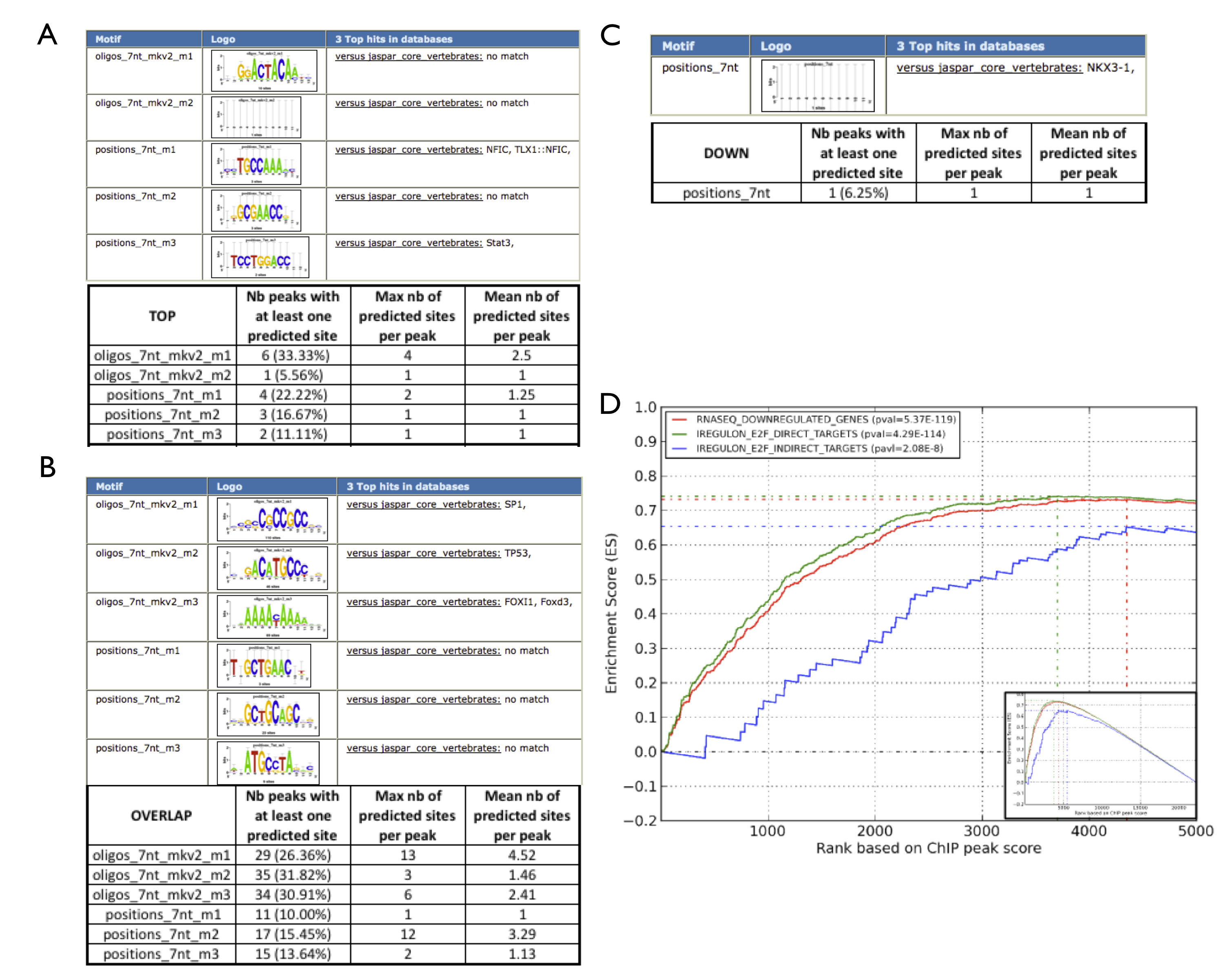

Supplement: Figure S6 — Validation of predicted regulons. A–C. PeakMotifs results. (A) Results of peakMotifs when applied on peaks near genes that are NOT predicted as direct p53 targets by iRegulon. On this set the p53 motif is not found. (B) Results on the ChIP peaks of up-regulated genes that are also direct targets. On this set of peaks the p53 motif is clearly found. (C) Results on the peaks near down-regulated genes, again not finding the p53 motif. D. GSEA results validating the iRegulon E2F predicted targets with E2F1ChIP-Seq results. Both the total set of down-regulated genes and the predicted E2F direct targets are highly enriched. E2F ChIP-Seq data in the same MCF-7 cell line were downloaded as fastq files from ENCODE. The sequences were mapped to hg19 using same mapping parameters as for p53 ChIP-Seq experiments and the bam files of the replicates were merged with samtools. See Experimental Procedures for the description of the peak calling and ranking of the genes. ENCODE Ids: wgEncodeYaleChIPseqRawDataRep1Mcf7Hae2f1, wgEncodeYaleChIPseqRawDataRep2Mcf7Hae2f1, wgEncodeYaleChIPseqRawDataRep1Mcf7Input, wgEncodeYaleChIPseqRawDataRep2Mcf7Input. (TIF) [file pcbi.1003731.s006.tif]

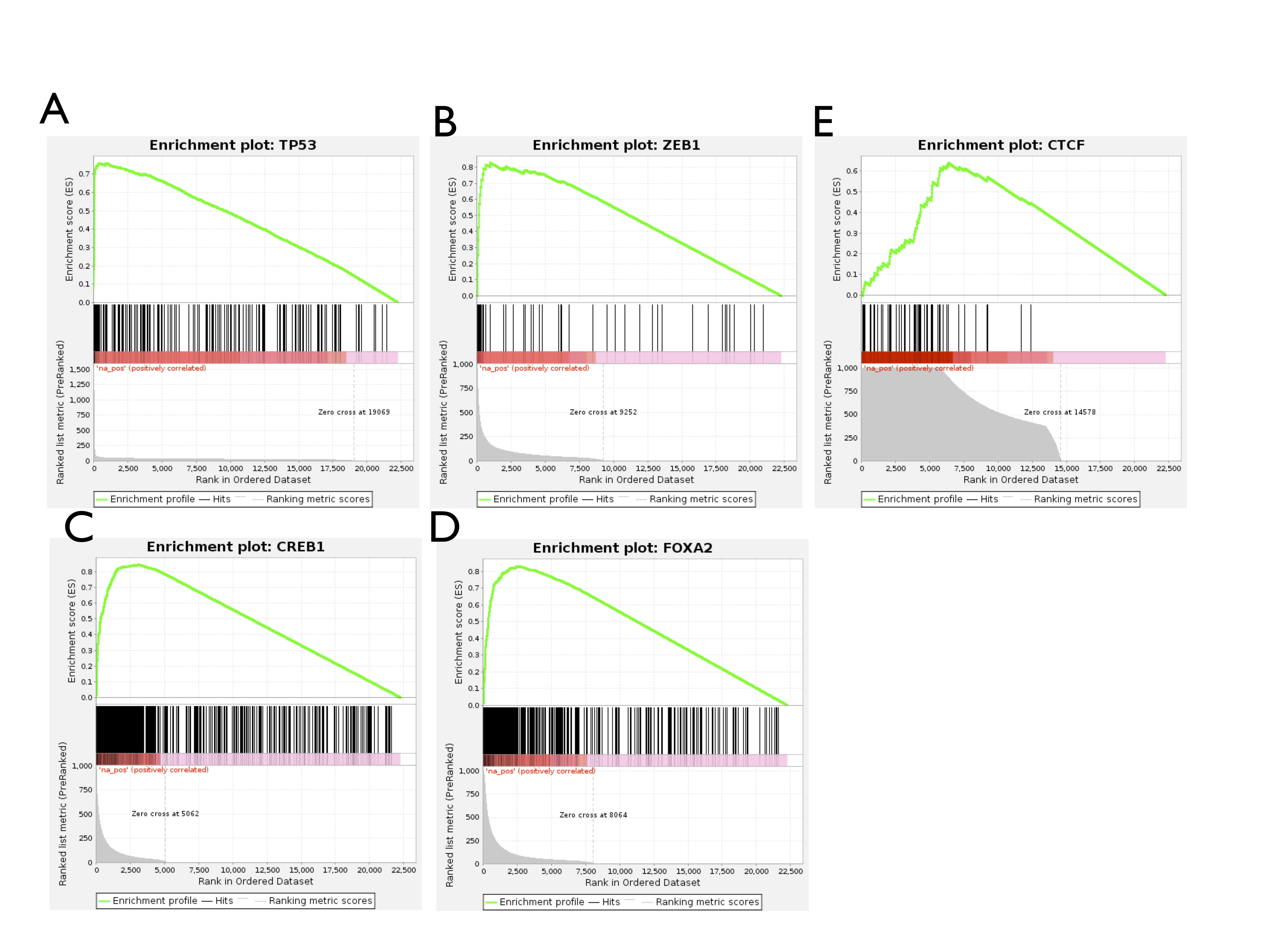

Supplement: Figure S7 — Gene Set Enrichment Analysis (GSEA) on GeneSigDB Meta-regulons. A. p53 meta-regulon (188 genes, min 3 signatures) is found positively enriched by GSEA on the preranked list of genes weighted by our in house p53 ChIP-Seq peak scores with a NES of 3.01, Nominal p-value = 0, FDR q-value = 0, leading edge at 890th rank of the signature. (B–E) GeneSigDB meta-regulon for TFs found enriched in ENCODE ChIP-Seq data using GSEA with 516/827 gene sets that passed the gene set size filters (min = 15, max = 1000) and corresponding to 78 TFs used in ENCODE ChIP-Seq datasets. B. ZEB1 meta-regulon (46 genes) is found positively enriched with a NES of 1.24, Nominal p-value = 0.001, FDR q-value = 0.918, leading edge at 950th rank of the signature. C. CREB1 meta-regulon (512 genes) is found positively enriched with a NES of 1.07, Nominal p-value = 0, FDR q-value = 1, leading edge at 3069th rank of the signature. D. FOXA2 meta-regulon (410 genes) is found positively enriched with a NES of 1.21, Nominal p-value = 0, FDR q-value = 0.191, leading edge at 3069th rank of the signature. E. CTCF meta-regulon (57 genes) is found positively enriched with a NES of 1.48, Nominal p-value = 0, FDR q-value = 0.11, leading edge at 6353th rank of the signature. Signature IDs are wgEncodeHaibTfbsGm12878Zeb1sc25388V0416102PkRep2 (B), wgEncodeHaibTfbsEcc1Creb1sc240V0422111PkRep2 (C), wgEncodeHaibTfbsA549Foxa2V0416102Etoh02PkRep1 (D), and wgEncodeSydhTfbsK562CtcfbIggrabPk (E). (TIF) [file pcbi.1003731.s007.tif]

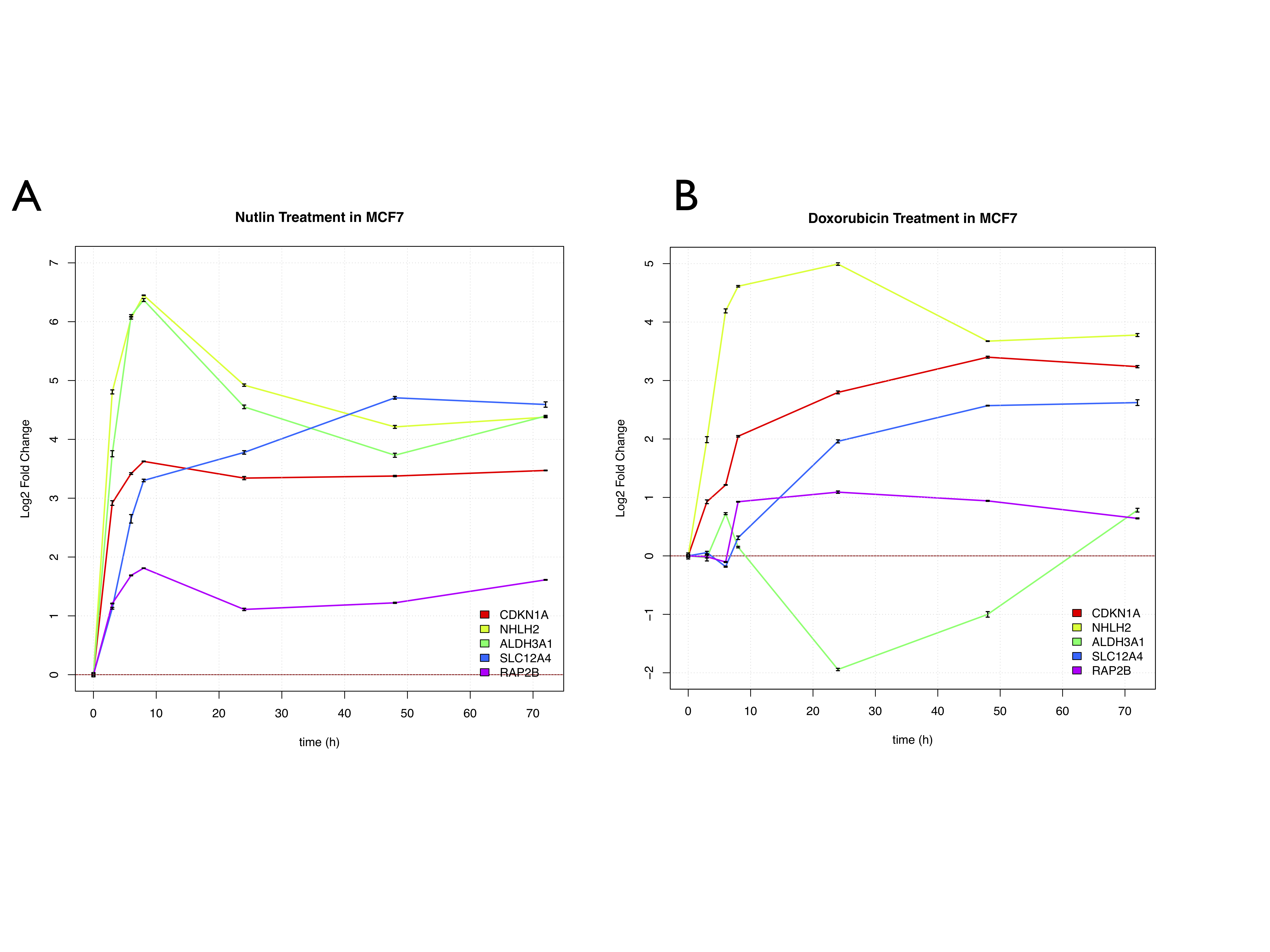

Supplement: Figure S8 — Time-course experiments by RT-qPCR. mRNA levels in log2FC of p53 target genes in MCF-7 cells after stimulation with 10 mM Nutlin3a (A) or 1 hour pulse of 5 mM Doxorubicin (B). (TIF) [file pcbi.1003731.s008.tif]
